# Sema4A Protects Against Muscle Atrophy and Promotes Repair by Regulating Intracellular Metabolic Signalling

**DOI:** 10.1002/jcsm.70315

**Published:** 2026-05-29

**Authors:** Caiyun Wang, Honghong Chen, Lingjie Shen, Shenhan Xu, Jie Xu, Qiuyuan Huang, Weiheng Zhang, Chengshou Lin, Jun Chang, Xinhua Liu, Wugui Chen

**Affiliations:** ^1^ Department of Orthopaedics Mindong Hospital, Fujian Medical University Fujian China; ^2^ Pharmacophenomics Laboratory, Human Phenome Institute Fudan University Shanghai China; ^3^ School of Sports Science and Engineering East China University of Science and Technology Shanghai China

**Keywords:** FoxO3a, muscle atrophy, muscle repair, Plexin B2, Semaphorin 4A

## Abstract

**Background:**

Skeletal muscle atrophy is a debilitating condition associated with diverse diseases and clinical interventions. Although triggers such as glucocorticoid excess or direct muscle injury disrupt the balance between protein homeostasis and tissue repair, the upstream molecular signals that actively preserve muscle integrity remain largely unknown. Semaphorin 4A (Sema4A) is a Class IV transmembrane semaphorin traditionally recognized for its roles in neural development and immune regulation; however, its function in skeletal muscle maintenance has not been explored.

**Methods:**

We first examined Sema4A expression in established models of muscle catabolism. Subsequently, adeno‐associated virus (AAV)‐mediated Sema4A restoration was employed to evaluate its therapeutic potential in both dexamethasone‐induced atrophy and acute injury mouse models. Mechanistic studies were performed in C2C12 myotubes using gain‐ and loss‐of‐function approaches.

**Results:**

Sema4A expression was significantly downregulated under atrophic conditions (*p* < 0.05). Restoration of Sema4A effectively attenuated dexamethasone‐induced muscle loss (4.766 vs. 5.075 mg/g B.W., *p* = 0.0414) and accelerated functional recovery (6.219 × 10^−2^ vs. 7.124 × 10^−2^ N/g B.W., *p* = 0.0422). We identified Plexin B2 (Plxnb2) as the functional receptor mediating these effects. At the molecular level, Sema4A signalling suppressed FoxO3a nuclear translocation (FoxO3a positive cells: vector‐Dex 27.99% vs. OE‐Sema4A‐Dex 12.49%, *p* < 0.05), thereby inhibiting the expression of key atrogenes, while simultaneously reactivating the PI3K‐AKT‐mTOR anabolic pathway. This reprogramming of intracellular metabolic signalling was further associated with the establishment of a reparative immune microenvironment, a process potentially modulated by muscle‐derived factors such as Gdf15.

**Conclusions:**

Our study identifies Sema4A as a novel protective regulator that mitigates muscle atrophy and enhances regeneration through a dual mechanism: restoring intracellular anabolic signalling and fostering a proregenerative immune niche. These findings highlight Sema4A as a promising therapeutic target for the treatment of muscle‐wasting disorders.

## 1 Introduction

Skeletal muscle, the most abundant and plastic tissue in the human body, accounts for approximately 40% of body weight and 50%–75% of total body protein [1, 2, 3, 4, 5]. Beyond its mechanical role in movement, it is essential for respiration, energy metabolism and the maintenance of systemic homeostasis [4]. Muscle atrophy arises from diverse causes, including denervation, disuse, fasting and severe systemic illnesses such as sepsis, organ failure, Cushing's syndrome and major trauma, all of which disrupt the delicate balance between protein synthesis and degradation [6]. This imbalance is also a hallmark of cancer cachexia, heart disease, obesity and aging‐related sarcopenia [7].

At the molecular level, diverse atrophic stimuli converge on the activation of common protein breakdown pathways, notably the ubiquitin‐proteasome system (UPS) and autophagy [6]. Sustained UPS activation indicates a shared transcriptional program that promotes proteolysis while suppressing synthesis [8, 9]. Glucocorticoids are key mediators of muscle wasting under chronic stress. By binding to intracellular receptors and translocating to the nucleus, they regulate gene expression, in part through forkhead box protein O (FoxO) transcription factors that upregulate E3 ubiquitin ligases such as Atrogin‐1 and MuRF1 [10].

Skeletal muscle possesses a robust regenerative capacity, primarily driven by muscle stem cells (satellite cells) that activate, proliferate and differentiate to repair damage [11]. The immune microenvironment plays an indispensable role in this process [12]. Macrophages undergo a tightly regulated phenotypic transition: Early proinflammatory macrophages clear debris and activate satellite cells, later switching to a restorative phenotype that supports myogenic differentiation and angiogenesis, a transition modulated by metabolic reprogramming [13].

Originally identified as axon guidance molecules, semaphorins are now recognized as versatile regulators in diverse physiological and pathological contexts, including immune regulation [14, 15]. In skeletal muscle, research has largely centered on Sema3A, which participates in neuromuscular junction remodelling and myoblast fusion [16], and is induced by growth factors from anti‐inflammatory macrophages [17]. mTOR, a central metabolic regulator, can modulate semaphorin function, hinting at a link between semaphorins and cellular metabolism [18]. Sema4A, a Class IV semaphorin, is involved in neurodevelopment, angiogenesis and immune responses [19]. It binds to Neuropilin‐1 (Nrp1) and can inhibit AKT‐mTOR signalling while promoting FoxO3a nuclear localization [20]. Sema4A deficiency also sensitizes haematopoietic stem cells to inflammatory stress, impairing differentiation [21]. Despite these roles, its function in skeletal muscle remains unknown. Intriguingly, analysis of public GEO datasets revealed consistent downregulation of Sema4A in multiple muscle atrophy models, suggesting that its loss may be a critical event in disease progression.

In this study, using dexamethasone (Dex)‐induced atrophy and cardiotoxin (CTX)‐induced injury models, we identify Sema4A as a novel regulator of muscle homeostasis. We demonstrate that Sema4A activates Plexin B2‐dependent signalling to restrain FoxO3a activity and sustain mTOR signalling. This cell‐intrinsic regulation further promotes a reparative immune microenvironment, potentially mediated by Gdf15. Thus, Sema4A emerges as a key integrator of metabolic signalling and immune support, essential for maintaining muscle integrity in health and disease.

## 2 Methods

Detailed information is provided in Supporting Information.

### 2.1 Animals and Experimental Models

The Institutional Animal Care and Use Committee (IACUC) of Fudan University approved all animal procedures (2023JS068). We used male C57BL/6J mice aged 6 to 8 weeks for this study. The mice were housed in a controlled environment with a 12‐h light/dark cycle and had free access to standard food and water. To achieve in vivo overexpression of Sema4A, intramuscular injections were administered into the tibialis anterior (TA) muscles. An adeno‐associated virus (AAV)2/9 serotype vector carrying the Sema4A sequence (Hanbio, China) was injected at a dose of 1.2 × 10^10^ viral genomes per site. Control mice received an equivalent dose of a nontargeting scrambled AAV. Disease models were initiated 3 weeks postinjection to allow for sufficient viral expression.

#### 2.1.1 Acute Muscle Injury

Muscle damage was induced by intramuscular injection of 80‐μL cardiotoxin (CTX, 20 μM) into the TA muscle. Muscle samples were harvested 7 days postinjury.

#### 2.1.2 Dex‐Induced Atrophy

Mice were administered daily intraperitoneal injections of Dexamethasone (Dex, 20 mg/kg) or saline for 10 consecutive days. Body weights were monitored daily throughout the treatment period.

### 2.2 Grip Strength Assessment

Muscle strength was assessed using a Grip Strength Meter (Zhongshi Technology, Beijing). Mice were placed on a metal grid and allowed to grip with all four paws. The tail was gently pulled backward until the grip was released. Three trials were recorded per animal, and the final values were normalized to body weight.

### 2.3 Histological Analyses

Muscle specimens were embedded in OCT compound (Servicebio, China) and flash‐frozen in solid CO_2_. Transverse sections were cut at a thickness of 10 μm. Sections were then stained with haematoxylin and eosin (H&E) following standard protocols. Myofiber cross‐sectional area (CSA) was quantified using ImageJ software.

#### 2.3.1 Immunofluorescence (IF)

Frozen sections were fixed in 4% paraformaldehyde for 10 min, permeabilized with 0.25% Triton X‐100 and blocked with 10% goat serum. Primary antibodies were applied overnight at 4°C. Following this, sections were incubated with fluorophore‐conjugated secondary antibodies. Nuclei were counterstained with DAPI.

#### 2.3.2 Immunohistochemistry (IHC)

Paraffin‐embedded muscle sections were deparaffinized in xylene and rehydrated through ethanol gradients. Antigen retrieval was performed by heating slides in citrate buffer (Beyotime) at 96°C for 15 min. Immunostaining signals were detected using species‐specific HRP/DAB kits (Abcam). Images were acquired with a Zeiss Axio Imager microscope.

### 2.4 RNA‐Seq Analysis

Total RNA was extracted from frozen muscle samples (−80°C). RNA concentration and integrity were determined using a Qubit 4.0 fluorometer and a Qsep400 bioanalyzer, respectively. After converting mRNA to cDNA, libraries were prepared via standard procedures, including end repair and adapter ligation. Library quality was verified on an Agilent 2100 Bioanalyzer. Clean reads were generated by filtering raw data, and differentially expressed genes (DEGs) were identified using DESeq2. Functional enrichment analysis was conducted on the Metware Cloud platform (https://cloud.metware.cn). Data are provided in the Supporting Information.

### 2.5 Cell Culture and Treatment

C2C12 myoblasts (ATCC, CRL‐1772) were maintained in DMEM supplemented with 10% FBS and antibiotics. Differentiation into myotubes was induced at 80%–90% confluence using medium containing 2% horse serum. The differentiation medium was refreshed every 48 h, and experiments were performed on day 4. C2C12 myotubes were treated with Dex (150 μM; Meilunbio) for 48 h to induce atrophy. For mTOR inhibition, Rapamycin (RAPA, 250 nM; TargetMol) was added for the final 24 h of Dex treatment. To prepare conditioned medium (CM), the supernatant from treated myotubes was collected and filtered through a 0.22‐μm membrane to remove cellular debris.

### 2.6 Macrophage Culture and Differentiation

#### 2.6.1 Primary Bone Marrow‐Derived Macrophages (BMDMs)

Bone marrow cells were flushed from the tibias and femurs of 6‐week‐old C57BL/6 mice. After red blood cell lysis, the suspension was incubated for 6 h to remove adherent stromal cells. Nonadherent monocytes were collected and differentiated in RPMI 1640 supplemented with 10% FBS and 10‐ng/mL M‐CSF. The medium was refreshed on Days 3 and 5. On Day 6, cells were treated with C2C12 conditioned medium (diluted 1:1 with fresh medium) for 48 h. The supernatant was subsequently filtered through a 0.22‐μm membrane to obtain BMDM conditioned medium.

#### 2.6.2 THP‐1 Cell Differentiation

THP‐1 cells were differentiated by treatment with 100‐ng/mL PMA for 48 h. Following a PBS wash and a 48‐h rest in fresh medium, cells were incubated with C2C12 conditioned medium (1:1 dilution) for another 48 h. The supernatant was collected and filtered to generate THP‐1 conditioned medium.

### 2.7 Small Interfering RNA (siRNA) Transfection

Cells were transfected with *Sema4A* siRNA or negative control siRNA (GenePharma, Shanghai) at 30%–50% confluence using Lipofectamine RNAiMAX (Thermo Fisher), following the manufacturer's protocol; 24 h posttransfection, the medium was replaced with fresh DMEM. Knockdown efficiency was validated by immunoblotting. siRNA sequences are listed in the Supporting Information.

### 2.8 Plasmid Transfection

For plasmid transfection, cells were transfected with 2 μg/mL of specific plasmids, including the empty vector, pLV3‐CMV‐Sema4A (mouse)‐3 × HA‐Puro and pLKO.1‐U6‐Plxnb2 (mouse)‐shRNA3‐mCherry‐Puro (MIAOLING, China), using Lipofectamine 2000 (Invitrogen). The culture medium was replaced 24 h after transfection, and cells were processed for downstream analyses.

### 2.9 Detection of Secreted Gdf15 (Methanol‐Chloroform Precipitation)

Secreted proteins in the culture medium were concentrated using the methanol‐chloroform precipitation method [22]. Briefly, an equal volume of methanol and one‐quarter volume of chloroform were added to the supernatant. The mixture was vortexed and centrifuged at 13000 ×*g* for 5 min to separate the phases. The upper aqueous layer and lower organic layer were discarded, retaining the interphase protein disc. One millilitre of methanol was added to wash the pellet, followed by vortexing and centrifugation at 13000 ×*g* for 10 min. The methanol was removed, and the pellet was air‐dried. The protein pellet was dissolved in 1× loading buffer, boiled at 95°C for 10 min and analysed via immunoblotting (details provided in the Supporting Information).

### 2.10 Statistical Analysis

Experiments were performed with at least three biological replicates. The exact sample size (*n*) for animal studies is specified in the corresponding figure legends. Data analysis was performed using GraphPad Prism 9 software. Results are presented as mean ± SEM. Differences between two groups were assessed using an unpaired two‐tailed Student's *t*‐test for single indicators, or multiple *t*‐tests with False Discovery Rate (FDR) correction for parallel indicators. Comparisons among three or more groups involving either single or multiple indicators were evaluated using a one‐way ANOVA followed by Dunnett's post hoc test. Time‐course data were analysed via a two‐way ANOVA followed by Sidak's multiple comparisons test. Statistical significance is defined as *p < 0.05, **p < 0.01 and ***p < 0.001.

## 3 Results

### 3.1 Sema4A Expression Is Downregulated in Multiple Models of Muscle Atrophy

**FIGURE 1:**
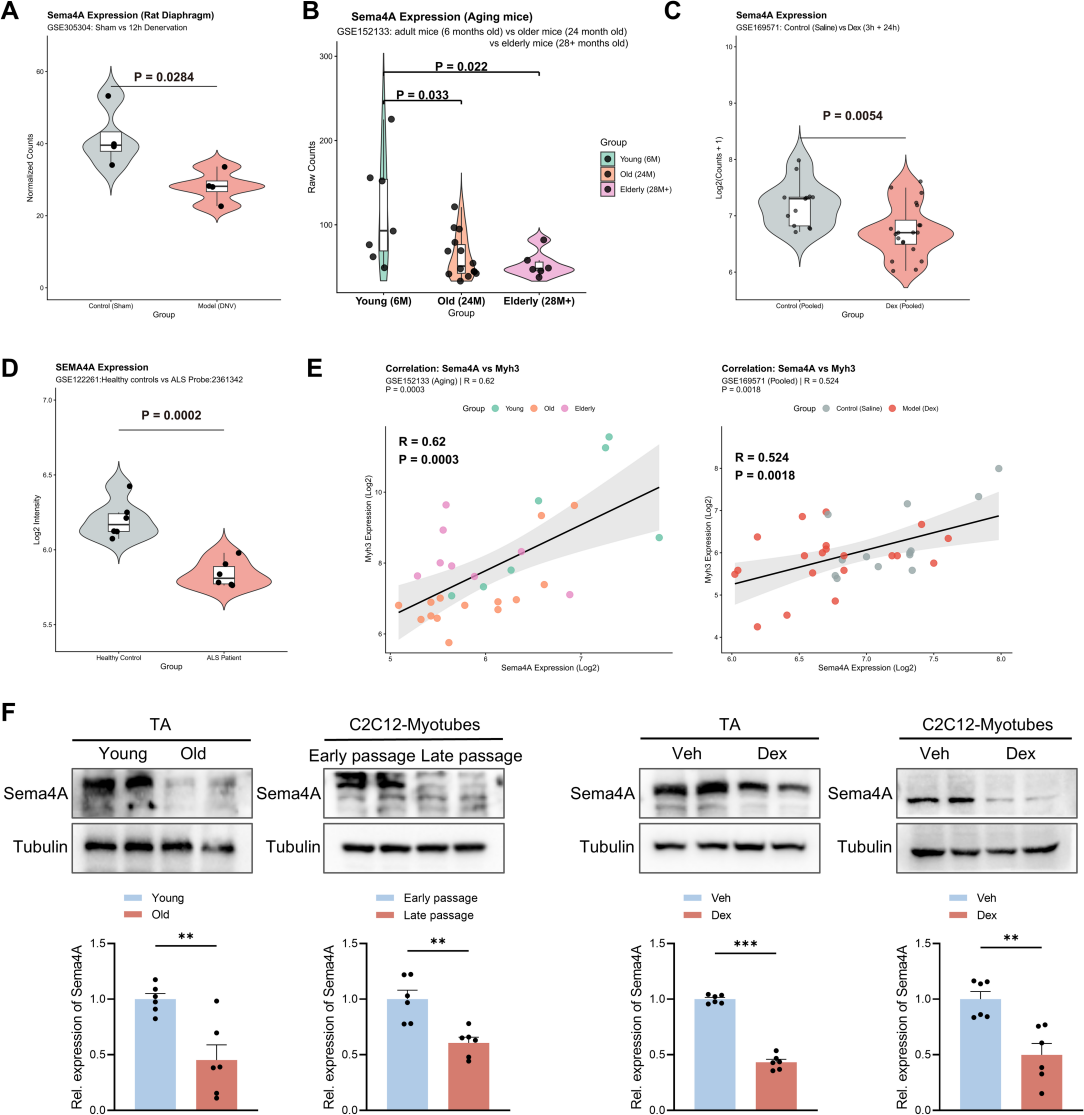
Sema4A is downregulated in muscle atrophy models. (A) RNA‐seq analysis of disuse‐induced diaphragmatic dysfunction and atrophy in rats (GSE305304). (B) Analysis of RNA‐seq data from tibialis anterior (TA) muscles of adult (6 months), older (24 months) and elderly (28+ months) mice (GSE152133). (C) RNA‐seq analysis of mouse quadriceps muscles harvested 3 or 24 h after intraperitoneal injection of dexamethasone sodium phosphate (20 mg/kg) (GSE169571). (D) Analysis of RNA‐seq data from primary muscle stem cells (myoblasts) extracted from deltoid muscle biopsies of amyotrophic lateral sclerosis (ALS) patients (GSE122261). (E) Correlation analysis between *Sema4A* and the myogenic regeneration marker *Myh3* within GSE152133 and GSE169571 datasets. (F) Validation of Sema4A protein expression in in vivo and in vitro models of aging (18‐month TA muscles and replicative senescence) and Dex‐induced atrophy (Dex‐treated TA muscles and C2C12 myotubes) (*n* = 6). Data are presented as mean ± SEM ** *p <* 0.01; ***p < 0.001. Statistical significance was determined using an unpaired two‐tailed *t*‐test.

To uncover novel regulators of muscle atrophy, we first conducted a bioinformatic screen using public transcriptomic datasets (GEO) from diverse atrophy models, including denervation, aging and glucocorticoid treatment. This analysis revealed a consistent downregulation of Sema4A across these distinct catabolic conditions (Figure 1A–C). The clinical relevance of this finding was supported by analysis of primary myoblasts from Amyotrophic Lateral Sclerosis (ALS) patients, which showed significantly lower SEMA4A expression compared to healthy controls (Figure 1D). Furthermore, in murine atrophy datasets (GSE152133 and GSE169571), Sema4A levels strongly correlated with the expression of the regeneration marker Myh3, suggesting a link between its loss and impaired muscle repair (Figure 1E).

We next experimentally validated these findings in a panel of in vivo and in vitro atrophy models. In aged (18‐month‐old) mice, Sema4A protein was markedly reduced in both TA and soleus (SOL) muscles, a decline also observed in senescent C2C12 myotubes (Figures 1F; S1A). Similarly, Sema4A was suppressed in a hindlimb unloading model of disuse atrophy (Figure S1B). Consistent with the bioinformatic prediction, dexamethasone‐induced atrophy led to a pronounced decrease in Sema4A (Figure 1F). Finally, oxidative stress modelled by hydrogen peroxide (H_2_O_2_) treatment also downregulated Sema4A in myotubes (Figure S1C). Taken together, these results identify reduced Sema4A expression as a recurrent feature of skeletal muscle atrophy across diverse conditions.

### 3.2 Sema4A Promotes Myogenic Differentiation and Myotube Formation in C2C12 Cells

**FIGURE 2:**
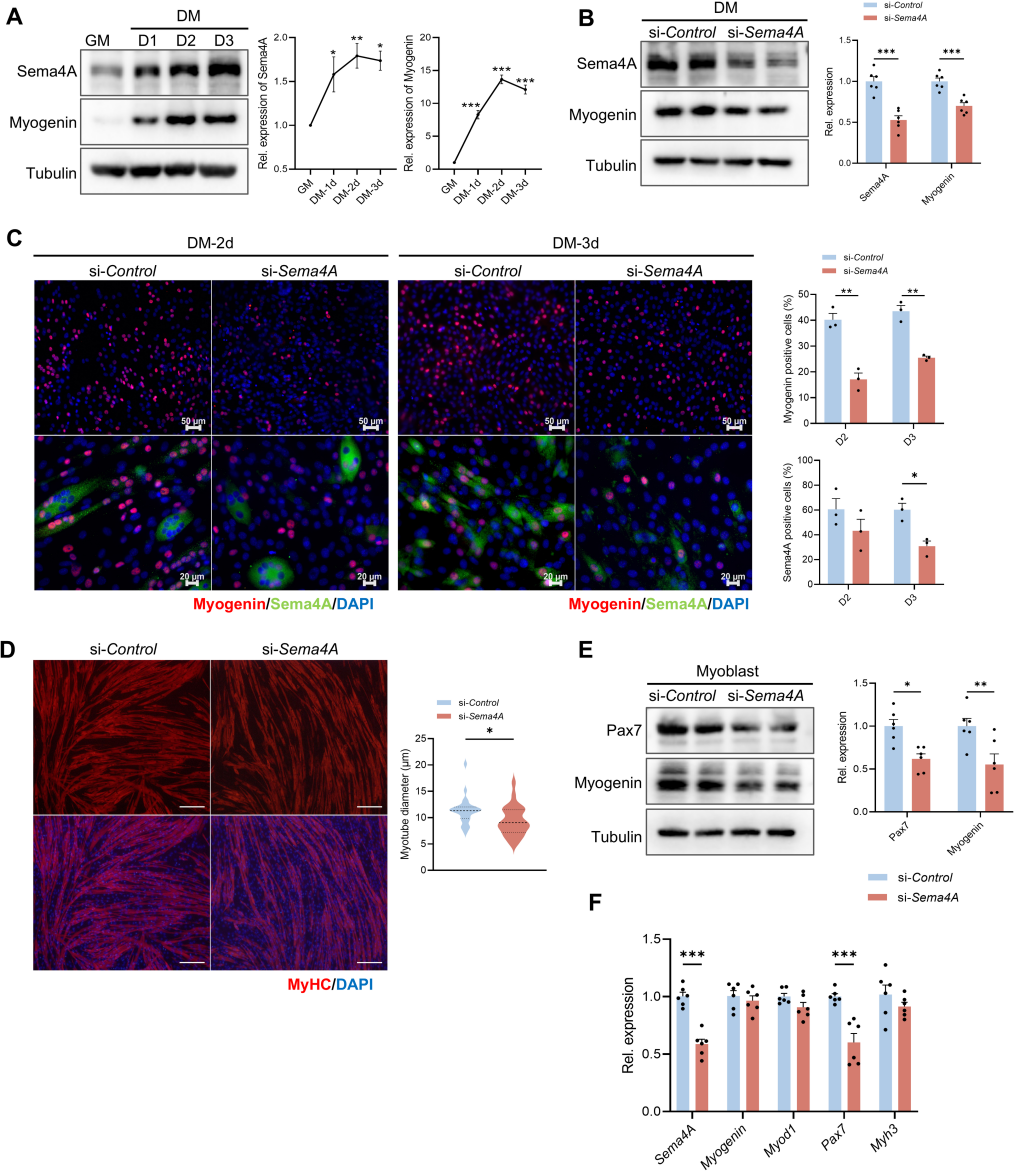
Sema4A deficiency impairs C2C12 myoblast differentiation. (A) Western blot analysis of Sema4A and myogenin expression in C2C12 cells during differentiation (*n* = 3). (B) Western blot analysis of Sema4A and myogenin in C2C12 cells transfected with *Sema4A* siRNA after 3 days of differentiation (*n* = 6). (C) Representative immunofluorescence images of Sema4A and myogenin in C2C12 myotubes at Day 2 and Day 3 of differentiation. Quantification of Sema4A intensity and the percentage of myogenin‐positive cells (*n* = 3). Scale bar: 50 and 20 μm. (D) Representative MyHC immunofluorescence images of C2C12 myotubes following *Sema4A* knockdown. Quantification of myotube diameter is shown. Scale bar: 100 μm. *n* = 25 myotubes per condition. (E) Western blot analysis of Pax7 and myogenin in C2C12 myoblasts transfected with *Sema4A* siRNA (*n* = 6). (F) Relative mRNA expression of indicated muscle regeneration genes determined by RT‐qPCR in C2C12 myoblasts transfected with *Sema4A* siRNA (*n* = 6). *p < 0.05; ** *p <* 0.01; ***p < 0.001. Data are presented as mean ± SEM. Statistical significance between two groups was determined by an unpaired two‐tailed Student's *t*‐test or multiple t‐tests for parallel indicators. Comparisons between differentiation time points and the Day 0 control group were evaluated using a one‐way ANOVA followed by Dunnett's post hoc test.

During the differentiation of C2C12 myoblasts, we observed a time‐dependent increase in Sema4A expression following the switch from growth medium (GM) to differentiation medium (DM) (Figure 2A). To investigate its functional role, we performed siRNA‐mediated knockdown of Sema4A. This intervention significantly attenuated the induction of myogenin, a key transcriptional regulator of differentiation, with the most pronounced effect seen at Day 2 (Figure 2B,C). By Day 3, although myogenin expression remained markedly impaired, the relative magnitude of this suppression was reduced, suggesting that Sema4A is strictly required for the timely initiation of the early differentiation program (Figure 2C). Consistent with this impaired early signalling, *Sema4A* knockdown also led to a clear functional deficit in terminal differentiation. Treated cells formed fewer myotubes, which exhibited significantly smaller diameters compared to control cells (Figure 2D). We further assessed the role of Sema4A in undifferentiated myoblasts. Its silencing resulted in a marked decrease in the protein abundance of both Pax7 (a marker of myogenic progenitors) and myogenin (Figure 2E). Notably, this reduction occurred without a proportional downregulation at the mRNA level for several myogenic factors (Figure 2F). This dissociation between transcript and protein levels suggests that Sema4A likely supports myogenesis through posttranscriptional mechanisms, such as regulating translation or protein stability, rather than solely via transcriptional control.

### 3.3 Sema4A Enhances Skeletal Muscle Regeneration Following Cardiotoxin‐Induced Injury

**FIGURE 3:**
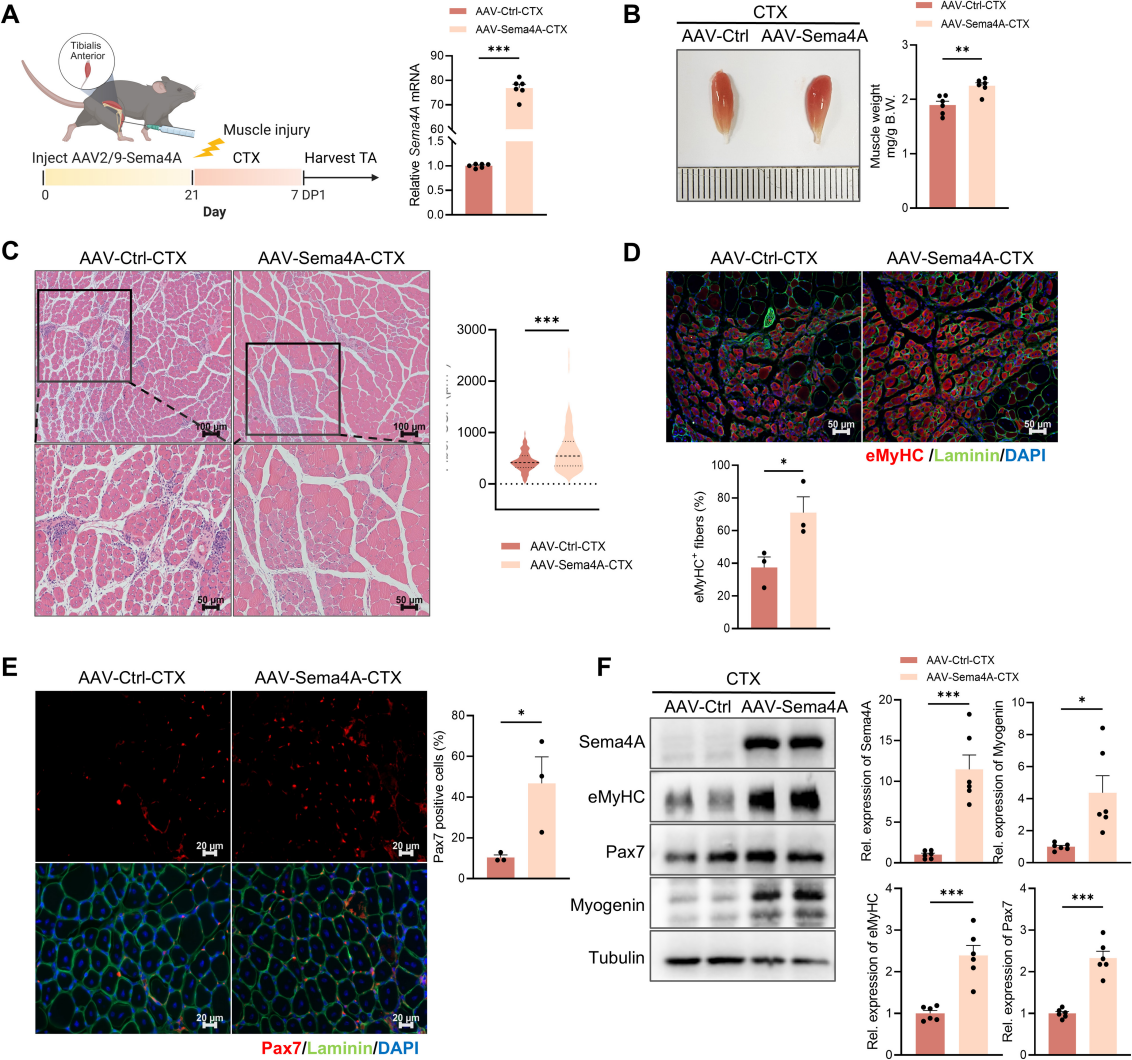
Sema4A promotes regeneration in CTX‐injured muscle. (A) Schematic illustration of the cardiotoxin (CTX)‐induced muscle injury model. Relative mRNA expression of *Sema4A* in TA muscles determined by RT‐qPCR (*n* = 6). (B) Representative images of TA muscles from AAV‐Ctrl and AAV‐Sema4A mice following CTX injection. TA muscle weight was normalized to body mass (*n* = 6). (C) Representative H&E staining images and quantification of CSA in TA muscle sections. Scale bars: 100 and 50 μm. 30–40 myofibers were analysed per image. (D) Representative immunofluorescence staining for eMyHC (red), Laminin (green) and DAPI (blue). Quantification of the percentage of eMyHC‐positive myofibers (*n* = 3). Scale bars: 50 μm. (E) Representative immunofluorescence staining for Pax7 (red), Laminin (green) and DAPI (blue). Quantification of the percentage of Pax7‐positive cells (*n* = 3). Scale bars: 20 μm. (F) Western blot analysis of eMyHC, Pax7 and myogenin expression in TA muscles (*n* = 6). *p < 0.05; ** *p <* 0.01; ***p < 0.001. Data are presented as mean ± SEM. Statistical significance was determined using an unpaired two‐tailed *t*‐test for two‐group comparisons.

To assess the role of Sema4A in muscle regeneration in vivo, we overexpressed Sema4A in mouse TA muscles via AAV2/9 delivery. Notably, 21 days after AAV injection under basal conditions, although body weight, functional grip strength and myofiber CSA remained largely unchanged, Sema4A overexpression significantly increased the frequency of centralized nuclei and embryonic myosin heavy chain (eMyHC, encoded by *Myh3*)‐positive fibres (Figure S2A–C). Consistent with these morphological signs of active remodelling, molecular analyses revealed that Sema4A significantly upregulated the protein levels of key myogenic transcription factors, including Pax7 and myogenin, along with an induction of myogenic gene transcription (Figure S2D,E). We then induced muscle injury by CTX injection and analysed regeneration 7 days later (Figure 3A). qPCR confirmed efficient and sustained *Sema4A* overexpression at this time point (Figure 3A). Muscles overexpressing Sema4A showed a modest increase in weight compared to controls (Figure 3B). Histological analysis revealed improved regeneration, characterized by reduced inflammatory infiltration and a greater CSA of newly formed myofibers in H&E‐stained sections (Figure 3C). Immunofluorescence for eMyHC indicated a significantly stronger regenerative response in the injured area of the Sema4A‐overexpressing group (Figure 3D). This was further supported by a marked increase in Pax7‐positive satellite cells (Figure 3E) and enhanced myogenin expression (Figure S2F). At the molecular level, Sema4A overexpression increased the protein abundance of key regenerative markers, including eMyHC, Pax7 and myogenin (Figure 3F), and upregulated the transcription of a broader set of myogenic factors (Figure S2G). Together, these in vivo results demonstrate that Sema4A not only establishes a proregenerative baseline in unstressed muscle but also acts as a positive regulator of repair following acute injury.

### 3.4 Sema4A Attenuates Glucocorticoid‐Induced Muscle Atrophy via Modulation of FoxO3a and mTOR Signalling

**FIGURE 4:**
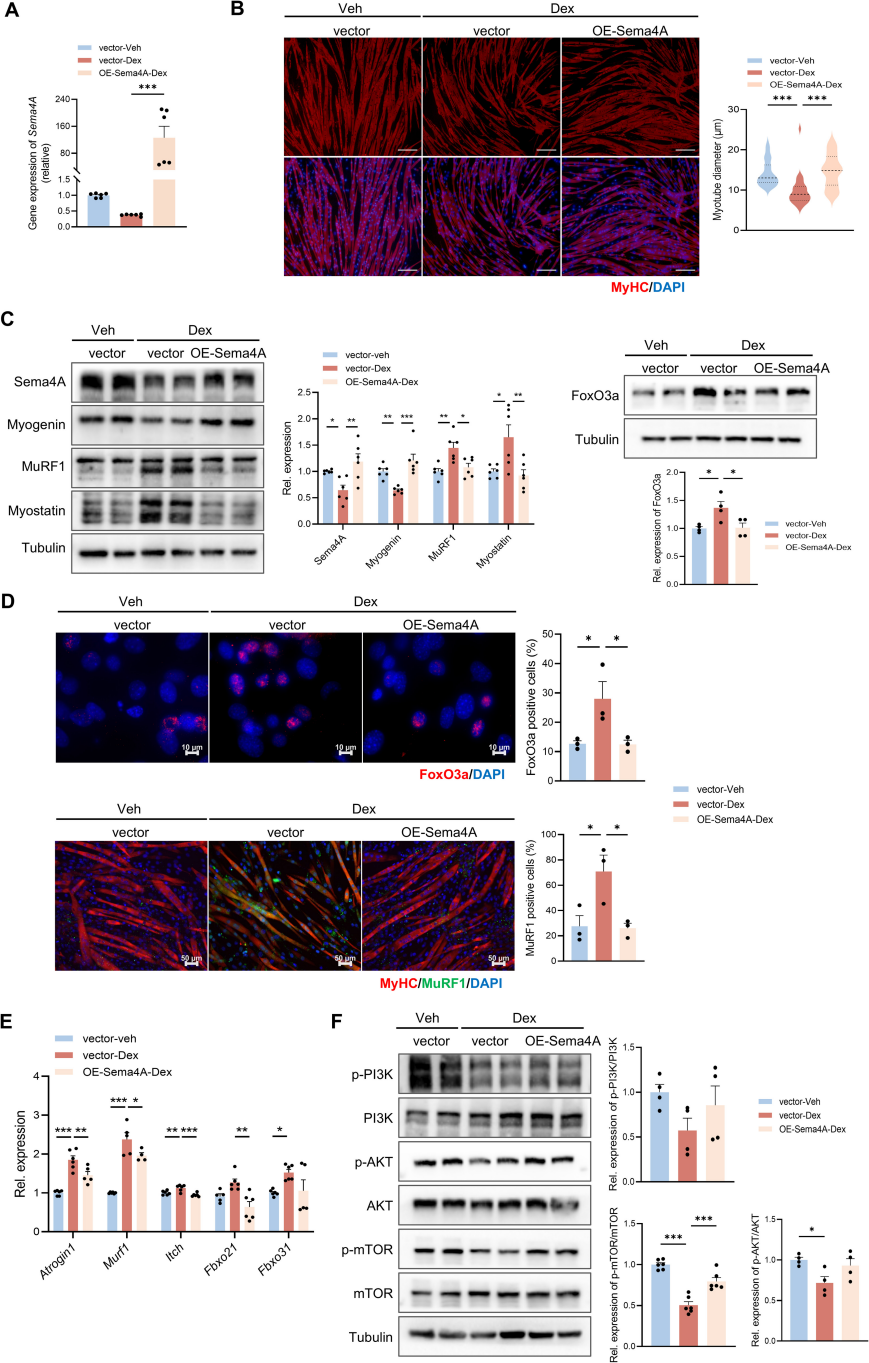
Sema4A overexpression rescues Dex‐induced muscle atrophy via FoxO3a regulation. (A) Relative mRNA expression of *Sema4A* in C2C12 myotubes treated with Dex (150 μM) in the presence or absence of Sema4A overexpression (OE‐Sema4A) (*n* = 6). (B) Representative MyHC immunofluorescence images of C2C12 myotubes under the indicated conditions. Quantification of myotube diameter is shown. Scale bar: 100 μm. 30 myotubes per condition. (C) Western blot analysis of Sema4A, myogenin, MuRF1, myostatin and FoxO3a expression in C2C12 myotubes under the indicated conditions. (*n* = 4–6). (D) Representative immunofluorescence images of FoxO3a (red, scale bars: 10 μm) and MyHC/MuRF1 (red/green, scale bars: 50 μm). Nuclei were stained with DAPI (blue). Quantifications indicate the percentage of FoxO3a‐ or MuRF1‐positive cells (*n* = 3). (E) Relative mRNA expression of indicated atrophy‐related genes in C2C12 myotubes under the indicated conditions. (*n* = 4–6). (F) Western blot analysis of the phosphorylation levels of PI3K, AKT and mTOR in C2C12 myotubes under the indicated conditions. (*n* = 4–6). *p < 0.05; ** *p <* 0.01; ***p < 0.001. Data are presented as mean ± SEM. Multiple groups were evaluated via one‐way ANOVA followed by Dunnett's post hoc test.

Following our in vivo findings, we investigated the mechanisms underlying the protective effects of Sema4A using an in vitro model of Dex‐induced atrophy in C2C12 myotubes. Dex treatment strongly suppressed Sema4A expression; based on dose‐response stability, 150 μM Dex was selected for subsequent experiments (Figure S3A). Overexpression of Sema4A (OE‐Sema4A) not only restored its own expression (Figure 4A) but also effectively rescued the myotube atrophy phenotype induced by Dex (Figure 4B). Furthermore, Sema4A overexpression in myoblasts prior to differentiation significantly improved the subsequent fusion index (Figure S3B).

At the molecular level, OE‐Sema4A reversed the Dex‐induced upregulation of the E3 ubiquitin ligase MuRF1 and the negative regulator myostatin (Figure 4C), while restoring the expression of key myogenic factors at both protein and mRNA levels (Figures 4C; S3C). We next investigated upstream regulatory events. Dex treatment increased the protein level of the proatrophy transcription factor FoxO3a, an effect that was markedly attenuated by OE‐Sema4A (Figure 4C). More importantly, immunofluorescence analysis revealed that OE‐Sema4A substantially reduced the nuclear accumulation of FoxO3a, effectively preventing its catabolic transcriptional activity (Figure 4D). Consequently, the expression of its downstream target MuRF1 was diminished (Figure 4D), and the transcriptional induction of a panel of atrogenes was broadly suppressed (Figure 4E). To assess the generality of this mechanism, we tested Sema4A's effect in an H_2_O_2_‐induced oxidative stress model (Figure S3D). Similarly, Sema4A overexpression significantly suppressed the induction of atrogenes and effectively protected against myotube atrophy under oxidative conditions (Figure S3E,F).

Given the critical balance between catabolic and anabolic signalling in muscle homeostasis, we examined whether Sema4A also influences the PI3K‐AKT‐mTOR anabolic axis. Western blot analysis confirmed that OE‐Sema4A significantly restored the phosphorylation of mTOR, which was otherwise inhibited by Dex (Figure 4F). In summary, these findings demonstrate that Sema4A counteracts glucocorticoid‐induced atrophy through a dual mechanism: inhibiting the nuclear translocation and activity of catabolic driver FoxO3a, while concurrently reactivating the anabolic mTOR signalling pathway.

### 3.5 AAV‐Mediated Sema4A Overexpression Alleviates Glucocorticoid‐Induced Muscle Atrophy and Preserves Regenerative Capacity

**FIGURE 5:**
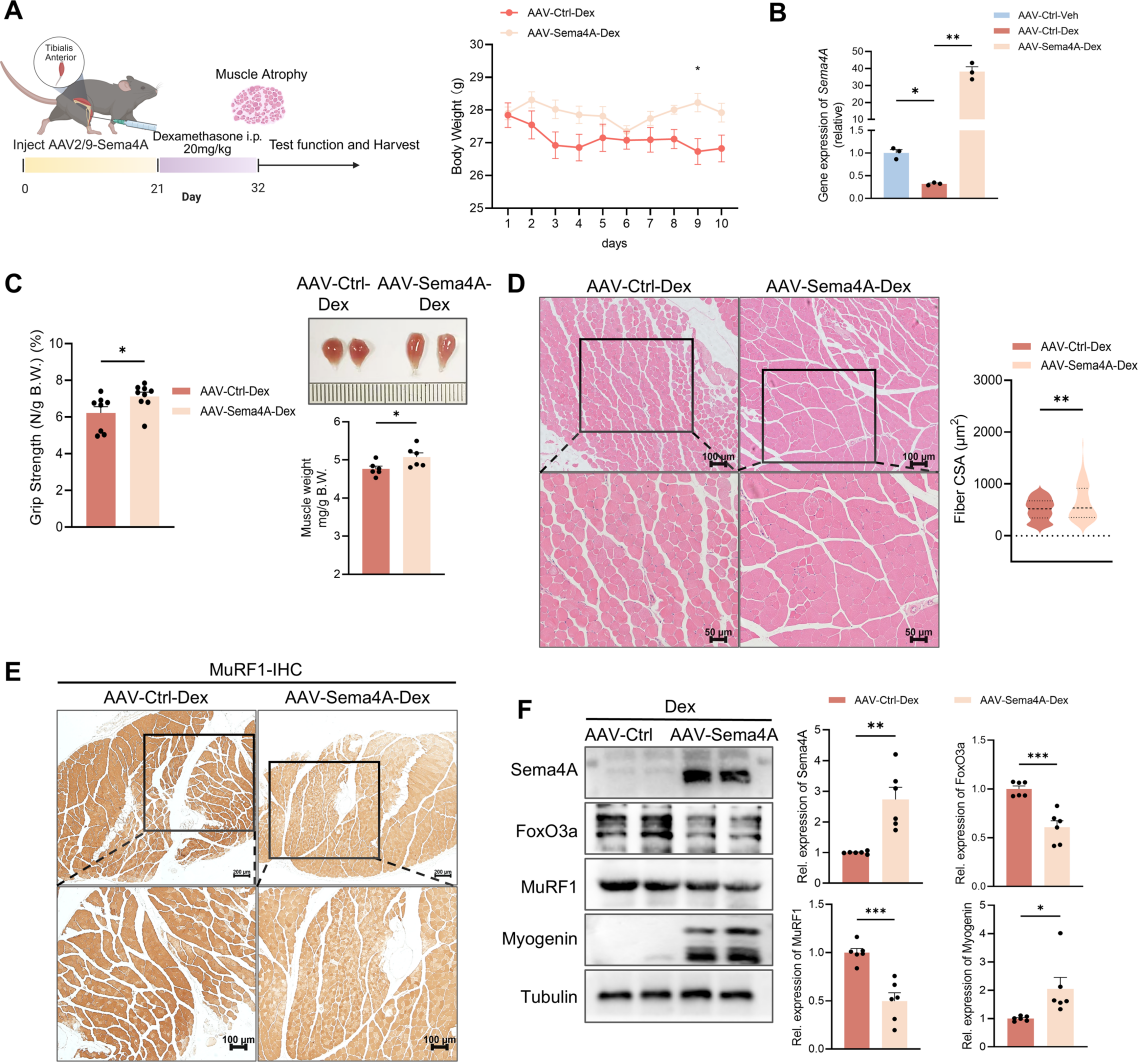
Sema4A overexpression protects against Dex‐induced skeletal muscle atrophy in vivo. (A) Schematic illustration of the Dex‐induced muscle atrophy model. Body weight changes monitored during the administration period (*n* = 14). (B) Relative mRNA expression of *Sema4A* in TA muscles determined by RT‐qPCR (*n* = 3). (C) Grip strength (normalized to body weight, N/g) in AAV‐Ctrl‐Dex and AAV‐Sema4A‐Dex mice (*n* = 8–9), and representative images of TA muscles. TA muscle weight was normalized to body mass (*n* = 6). (D) Representative H&E staining images and quantification of CSA in TA muscle sections (*n* = 3). Scale bars: 100 and 50 μm. 30–40 myofibers were analysed per image. (E) Representative immunohistochemistry images for MuRF1 in muscle cross sections. Scale bars: 200 and 100 μm. (F) Western blot analysis of Sema4A, FoxO3a, MuRF1 and myogenin expression in TA muscles from AAV‐Ctrl‐Dex and AAV‐Sema4A‐Dex mice (*n* = 6). *p < 0.05; ** *p <* 0.01; ***p < 0.001. Data are presented as mean ± SEM. Significance between two groups was determined by an unpaired two‐tailed Student's *t*‐test. Multiple groups were evaluated via one‐way ANOVA. Time‐course data were analysed via a two‐way ANOVA followed by Sidak's multiple comparisons test.

To evaluate the therapeutic potential of Sema4A in vivo, we overexpressed it in mouse TA muscles via intramuscular injection of AAV‐Sema4A. Notably, even under basal conditions, Sema4A overexpression downregulated both the mRNA and protein levels of key atrogenes after 21 days, suggesting a preventive role against muscle wasting (Figure S4A,B). We then induced muscle atrophy by intraperitoneal injection of Dex for 10 days (Figure 5A). Mice overexpressing Sema4A (AAV‐Sema4A‐Dex) exhibited mitigated weight loss compared to the control group (AAV‐Ctrl‐Dex), with a significant difference observed at Day 9 (Figure 5A). qPCR confirmed that AAV delivery effectively restored *Sema4A* transcript levels in atrophying muscle (Figure 5B). Functionally, Sema4A overexpression significantly improved the Dex‐induced deficits in grip strength and muscle mass (Figure 5C). Histological analysis revealed a partial rescue of the reduced myofiber CSA in the AAV‐Sema4A group (Figure 5D). Consistent with this phenotypic improvement, immunostaining showed that the strong MuRF1 signal induced by Dex was markedly reduced upon Sema4A overexpression (Figure 5E). At the molecular level, Sema4A overexpression decreased the protein abundance of FoxO3a and MuRF1 (Figure 5F) and suppressed the transcriptional upregulation of a broad set of atrogenes (Figure S4C).

Given the close link between atrophy and impaired regeneration, we assessed myogenic markers. Sema4A overexpression reversed the Dex‐induced decline in key myogenic factors (Figures 5F; S4D). This was further supported by the preservation of eMyHC‐positive myofibers (Figure S4E) and the maintained protein abundance of eMyHC and Pax7 (Figure S4F). In summary, AAV‐mediated Sema4A overexpression effectively counteracts glucocorticoid‐induced muscle atrophy in vivo through a synergistic mechanism involving both the suppression of the catabolic atrogene program at baseline and during stress and the active preservation of intrinsic myogenic capacity.

### 3.6 Sema4A Modulates the Immune Microenvironment to Facilitate Muscle Repair

**FIGURE 6:**
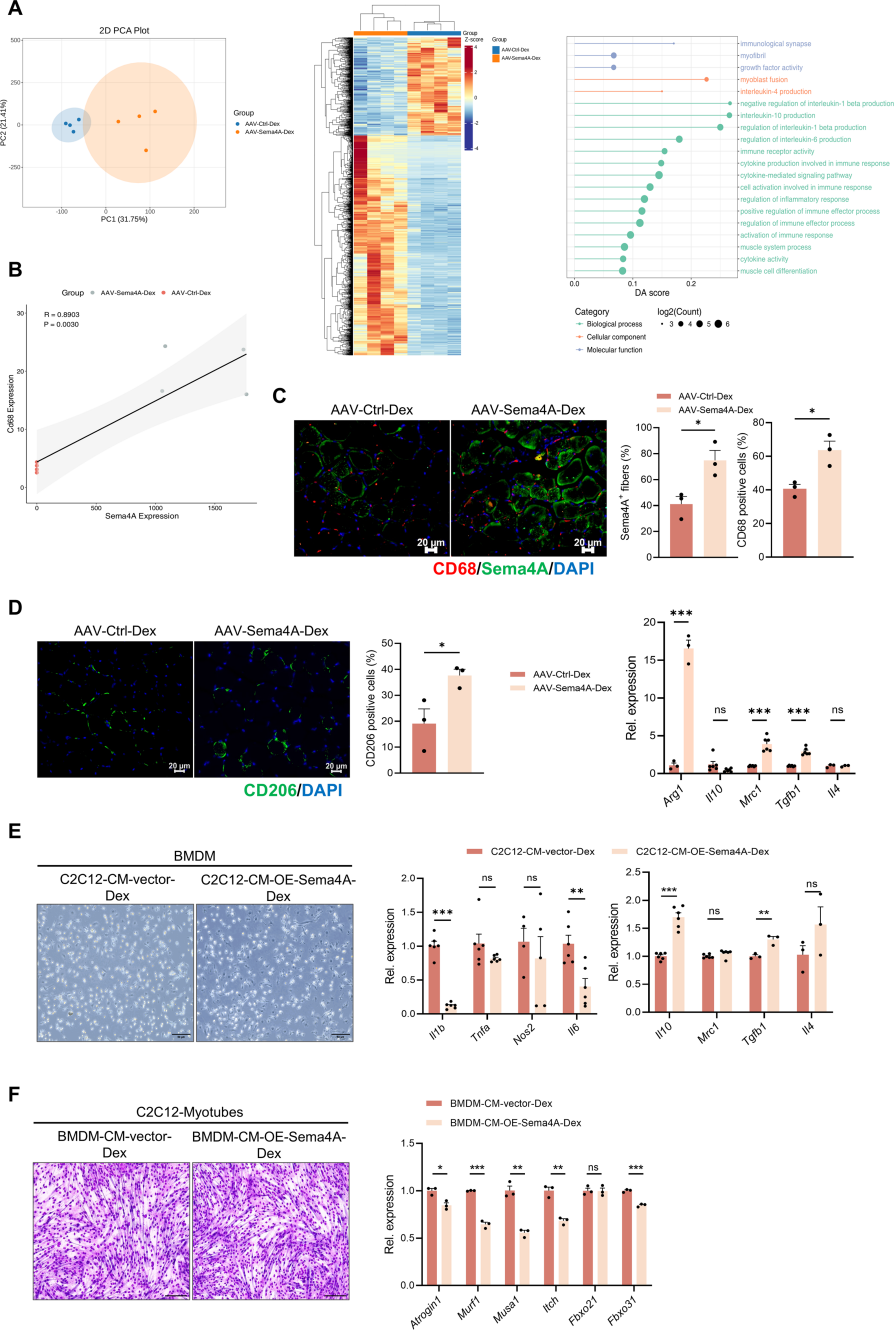
Sema4A facilitates a reparative immune microenvironment. (A) Principal component analysis (PCA) based on RNA‐seq data derived from TA muscles of AAV‐Ctrl‐Dex and AAV‐Sema4A‐Dex mice. Hierarchical clustering heat map of significantly differentially expressed genes (|Log2FC| > 1; adjusted *p*‐value < 0.05). Lollipop chart illustrating Gene Ontology (GO) enrichment analysis. (B) Correlation analysis between *Sema4A* and the macrophage marker *Cd68* in the RNA‐seq dataset. (C) Representative immunofluorescence staining for CD68 (red), Sema4A (green) and DAPI (blue). Quantification of Sema4A intensity in myofibers and the percentage of CD68‐positive cells (*n* = 3). Scale bars: 20 μm. (D) Representative images and quantification of CD206^+^ cells (green; scale bars, 20 μm), alongside relative mRNA expression of M2 markers in TA muscles from AAV‐Ctrl‐Dex and AAV‐Sema4A‐Dex mice (*n* = 3). (E) Representative morphological images and relative mRNA expression of M1/M2 markers in BMDMs following 48 h of incubation with C2C12‐derived CM (diluted 1:1 with fresh medium) (*n* = 3–6). (F) Representative morphological images and relative mRNA expression of atrophy‐related genes in C2C12 myotubes treated with CM from the pretreated BMDMs (*n* = 3). *p < 0.05; ** *p <* 0.01; ***p < 0.001. Data are presented as mean ± SEM. Significance between two groups was determined by an unpaired two‐tailed Student's *t*‐test, or multiple *t*‐tests for parallel indicators.

To explore the systemic effects of Sema4A beyond intrinsic myogenic regulation, we performed RNA sequencing (RNA‐seq) on TA muscles from AAV‐Sema4A‐Dex and AAV‐Ctrl‐Dex mice. Principal component analysis (PCA) revealed distinct transcriptomic clustering between the two groups. Subsequent differential expression analysis identified a set of genes significantly altered by Sema4A overexpression (|Log2FC| > 1, adj. *p <* 0.05) (Figure 6A). Gene Ontology (GO) enrichment revealed that these DEGs were strongly associated with immune processes, such as cytokine production and leukocyte activation, indicating a major impact of Sema4A on the muscle‐immune landscape (Figures 6A; S5A).

Correlation analysis within the RNA‐seq dataset showed a strong positive correlation between *Sema4A* and myogenic factors (*R* = 0.8432, *p* = 0.0085), consistent with our functional findings (Figure S5B). Notably, *Sema4A* expression also highly correlated with the pan‐macrophage marker *Cd68* (*R* = 0.8903, *p* = 0.0030) (Figure 6B). Immunofluorescence validation confirmed an increased abundance of CD68^+^ macrophages in Sema4A‐overexpressing muscle (Figure 6C). More importantly, Sema4A overexpression promoted a shift in macrophage polarization toward a restorative phenotype. We observed an increased frequency of CD206^+^ (M2‐like) macrophages and a transcriptional upregulation of multiple M2 markers (e.g.*, Arg1, Mrc1* and *Tgfb1*) (Figure 6D). Although changes in classical M1 markers (e.g.*, Il1b* and *Il6*) were variable, the key M1 marker *Nos2* was significantly downregulated, indicating a net shift toward an anti‐inflammatory state (Figure S5C). Interestingly, in uninjured muscle, Sema4A induced a unique immunomodulatory signature characterized by elevated *Tnfa* alongside a robust increase in *Arg1* (Figure S5D). Given that physiological levels of TNFα may facilitate early myoblast proliferation [23], this specific profile suggests that Sema4A primes the microenvironment toward a regenerative state, rather than inducing pathological inflammation.

We next asked whether muscle‐derived Sema4A could directly instruct macrophage plasticity. Conditioned media (CM) from Sema4A‐overexpressing myotubes induced an elongated, M2‐like morphology in BMDMs and shifted their transcriptional profile, decreasing M1 markers (*Il1b* and *Il6*) while increasing M2 markers (*Il10* and *Tgfb1*) (Figure 6E). This pretreated macrophage CM, in turn, promoted hypertrophy and suppressed atrogene expression in recipient myotubes (Figure 6F). Similar effects were observed using the human THP‐1 macrophage line (Figure S5E–H). Collectively, these findings establish a paracrine signalling loop wherein muscle‐derived Sema4A reprograms local macrophages toward a proregenerative phenotype, which subsequently provides trophic support to muscle cells, thereby integrating immune modulation with tissue repair.

### 3.7 Plexin B2 Mediates Sema4A Signalling in Skeletal Muscle

**FIGURE 7:**
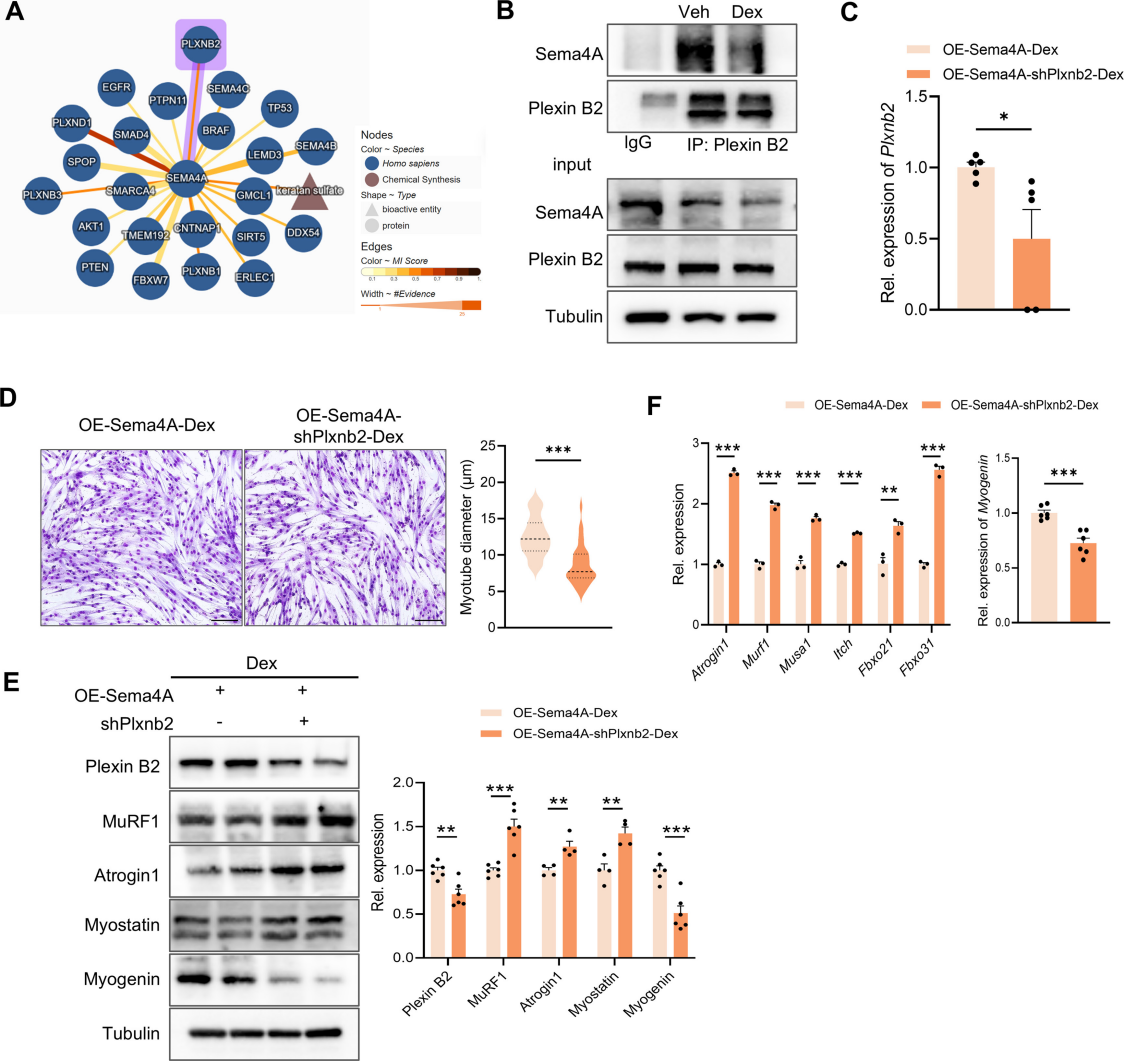
Plexin B2 mediates Sema4A signalling in skeletal muscle. (A) Protein‐protein‐interaction network analysis constructed using the IntAct database. (B) Coimmunoprecipitation (Co‐IP) assay verifying the interaction between Plexin B2 and Sema4A. (C) Relative mRNA expression of *Plxnb2* in Dex‐treated C2C12 myotubes following simultaneous OE‐Sema4A and Plxnb2 knockdown (shPlxnb2) (*n* = 3–5). (D) Representative Giemsa staining images of Dex‐treated C2C12 myotubes following simultaneous OE‐Sema4A and Plxnb2 knockdown. Quantification of myotube diameter is shown. Scale bar: 100 μm. 30–40 myotubes per condition. (E) Western blot analysis of Plexin B2, MuRF1, Atrogin1, myostatin and myogenin expression in Dex‐treated C2C12 myotubes following simultaneous OE‐Sema4A and Plxnb2 knockdown (*n* = 4–6). (F) Relative mRNA expression of indicated atrophy‐related genes and *myogenin* in Dex‐treated C2C12 myotubes following simultaneous OE‐Sema4A and Plxnb2 knockdown (*n* = 3–6). *p < 0.05; ** *p <* 0.01; ***p < 0.001. Data are presented as mean ± SEM. Significance between two groups was determined by an unpaired two‐tailed Student's *t*‐test, or multiple *t*‐tests for parallel indicators.

To identify the receptor responsible for transducing Sema4A signals in muscle, we performed a protein‐interaction network analysis. The IntAct database indicated Plexin B2 as a primary candidate binding partner for Sema4A (Figure 7A). We confirmed this interaction by coimmunoprecipitation (Co‐IP) in C2C12 myotubes, where endogenous Plexin B2 readily associated with Sema4A under control conditions. In the Dex‐induced atrophy model, this interaction was substantially weakened, consistent with the reduction in Sema4A expression (Figure 7B). To determine whether Plexin B2 is functionally required for Sema4A‐mediated protection, we silenced Plxnb2 with shRNA while overexpressing Sema4A in Dex‐treated myotubes (Figure 7C). Knockdown of Plexin B2 completely abolished the protective effect of Sema4A, as evidenced by severe myotube atrophy (Figure 7D). Moreover, the ability of Sema4A to suppress atrogenes (MuRF1, Atrogin‐1 and myostatin) and restore myogenic factors was lost when Plexin B2 was depleted (Figure 7E). These observations were consistent at the transcriptional level (Figure 7F). Together, these data demonstrate that Plexin B2 is an essential receptor for Sema4A in skeletal muscle and that the protective effects of Sema4A against glucocorticoid‐induced atrophy depend on Plexin B2‐mediated signalling.

### 3.8 Sema4A Promotes a Reparative Immune Microenvironment, Potentially Involving Gdf15

**FIGURE 8:**
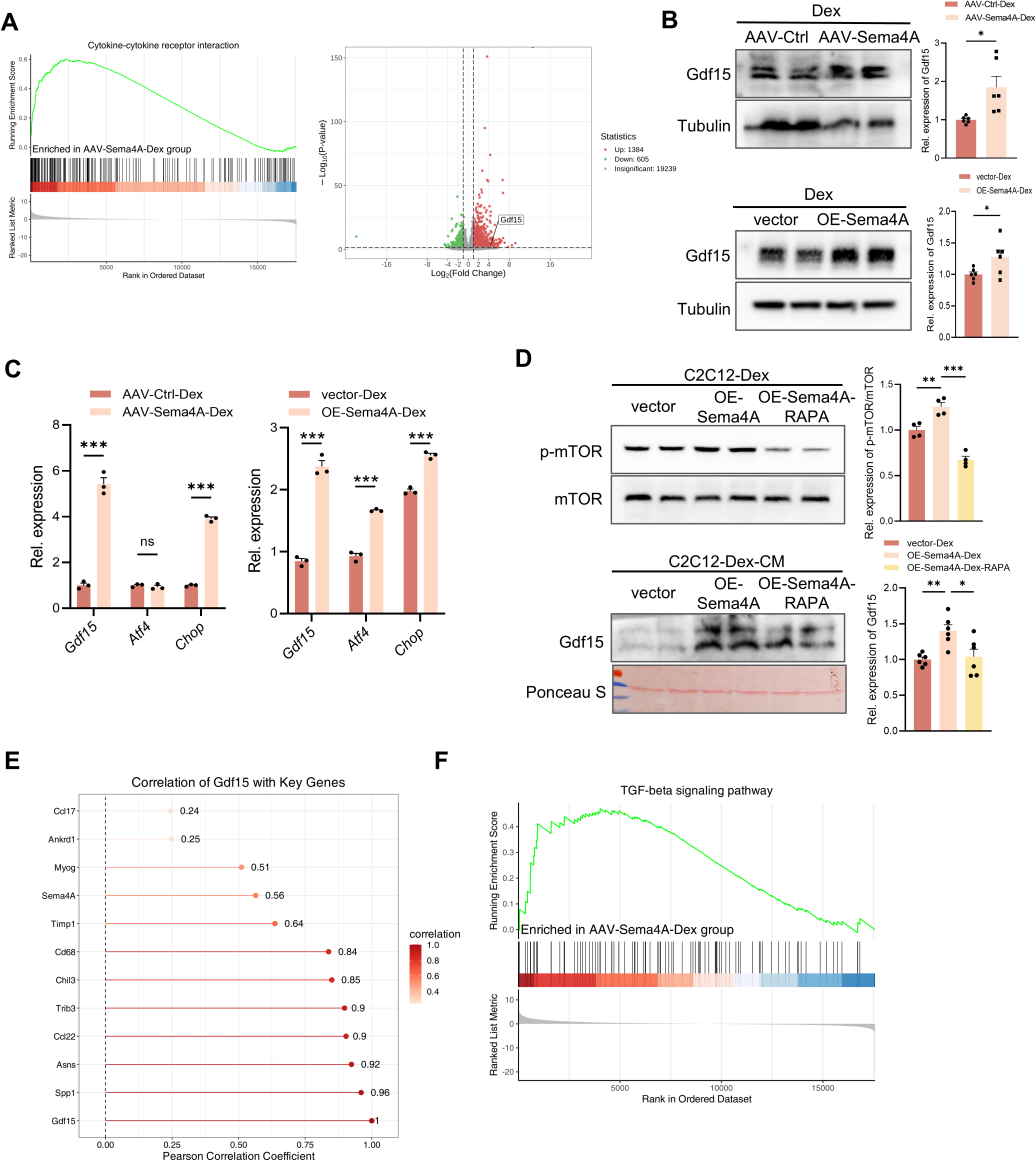
Sema4A‐driven Gdf15 supports a reparative immune microenvironment. (A) Gene set enrichment analysis (GSEA) of the ‘Cytokine‐cytokine receptor interaction’ pathway based on the RNA‐seq dataset. Volcano plot of the RNA‐seq data highlighting Gdf15. (B) Western blot analysis of Gdf15 expression in TA muscles and C2C12 myotubes (*n* = 6). (C) Relative mRNA expression of *Gdf15*, *Atf4* and *Chop* in TA muscles and C2C12 myotubes (*n* = 3). (D) Representative Western blot and quantification of secreted Gdf15 in the CM of Dex‐treated C2C12 myotubes, following Rapamycin (250 nM, 24 h) treatment. Ponceau S staining served as a loading control for CM proteins (*n* = 6). (E) Correlation analysis between *Gdf15* and *Sema4A*, stress markers (*Asns* and *Trib3*), recruitment signals (*Cd68* and *Spp1*) and M2 markers (*Timp1, Chil3, Ccl22, Myog* and *Ankrd1*). (F) GSEA of the TGF‐*β* signalling pathway. *p < 0.05; ** *p <* 0.01; ***p < 0.001. Data are presented as mean ± SEM. Significance between two groups was determined by an unpaired two‐tailed Student's *t*‐test, or multiple *t*‐tests for parallel indicators. Multiple groups were evaluated via one‐way ANOVA.

Having demonstrated that Sema4A activates PI3K‐AKT‐mTOR signalling through Plexin B2 and promotes M2 macrophage polarization, we next sought to identify the secretory mediators linking intramyocellular signalling to extracellular immune modulation. Gene Set Enrichment Analysis (GSEA) of our RNA‐seq data revealed a significant enrichment of ‘cytokine‐cytokine receptor interaction’ in Sema4A‐overexpressing muscle (Figure 8A). Within this signature, Gdf15, a stress‐responsive myokine with established immunomodulatory functions [24, 25, 26], emerged as a top candidate, showing a marked increase (Log2FC > 4) (Figure 8A). Interestingly, distinct regulatory mechanisms appeared to govern Gdf15 expression depending on the cellular context. Under basal conditions (without Dex), Sema4A overexpression upregulated *Gdf15* and *Tp53* (known as p53), whereas *Atf4* and C/EBP homologous protein (*Chop*) remained unchanged (Figure S6A). This suggests that in the absence of exogenous stress, Sema4A induces Gdf15 possibly via a p53‐dependent mechanism, likely serving as a physiological ‘priming’ signal rather than a maladaptive stress response. In the setting of Dex‐induced atrophy, where Sema4A restores mTOR activity (a known driver of Atf4 synthesis), we observed a robust increase in Gdf15 at both protein and mRNA levels (Figure 8B). Consistently, the expression of its upstream regulators, *Atf4* and *Chop*, was elevated in both tissue and cell models (Figure 8C). GSEA further supported the concomitant activation of p53 signalling, which may cooperate to amplify Gdf15 transcription (Figure S6B).

Importantly, Sema4A overexpression significantly increased Gdf15 secretion in the conditioned medium of Dex‐treated myotubes, an effect that was reversed by the mTOR inhibitor Rapamycin (Figure 8D). Molecularly, Rapamycin blunted the Sema4A‐induced upregulation of adaptive stress markers *Atf4* and *Chop*, leading to the reduction of Gdf15. Concurrently, *p53* levels were further elevated, reflecting a shift toward unresolved pathological stress upon disruption of the protective mTOR‐ATF4 axis (Figure S6C). Functionally, inhibiting mTOR impaired the capacity of Sema4A‐OE CM to drive M2 macrophage polarization, as evidenced by a reduction in elongated M2‐like cells and a transcriptional shift toward a proinflammatory profile (Figure S6D,E).

Correlation network analysis positioned Gdf15 within a functional interaction landscape, linking its expression not only to *Sema4A* itself, but also to stress markers (e.g., *Asns* and *Trib3*), macrophage recruitment signals (e.g., *Cd68* and *Spp1*) and key M2/repair‐associated genes (e.g., *Timp1, Chil3, Ccl22, Myog* and *Ankrd1*) (Figures 8E; S6F). This pattern suggests that Sema4A orchestrates an adaptive response wherein stress sensing drives Gdf15 secretion, which in turn facilitates macrophage recruitment and polarization toward an M2‐reparative phenotype. Consistently, related pathways such as TGF‐*β* signalling were also enriched, further corroborating a proregenerative microenvironment [27] (Figure 8F). In summary, these findings reveal that Sema4A regulates intracellular metabolic signalling, coupling muscle homeostasis with immune‐mediated mechanisms that partially contribute to injury repair, potentially involving Gdf15.

## 4 Discussion

**FIGURE 9:**
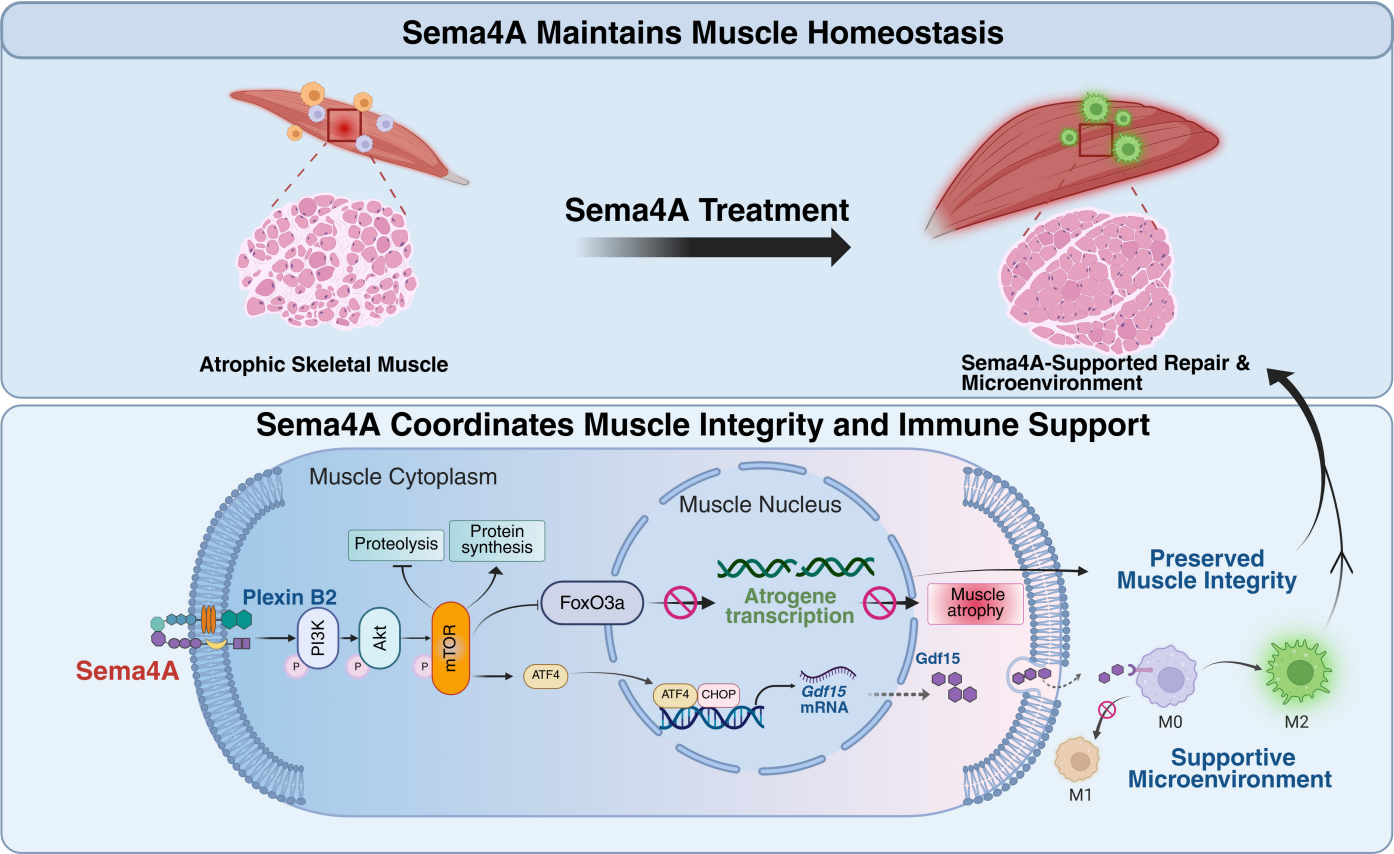
Schematic diagram depicting how Sema4A regulates intracellular metabolic signalling to maintain muscle homeostasis and promote repair.

Although the downstream effectors of muscle atrophy are well characterized, the upstream signals that actively coordinate protection and repair remain unclear. This study identifies Sema4A as a key regulator of muscle homeostasis, showing that its restoration reverses glucocorticoid‐induced atrophy and accelerates repair (Figure 9). Mechanistically, Sema4A signalling via Plexin B2 suppresses FoxO3a‐mediated transcription of atrogenes while concurrently reactivating the PI3K‐AKT‐mTOR pathway. This intracellular regulation further shapes a favourable immune microenvironment, partially involving paracrine factors such as Gdf15. Our findings establish Sema4A as a critical upstream signal that couples metabolic signalling to the preservation of muscle integrity.

Semaphorins are primarily known to regulate cell migration, macrophage polarization and cytokine release in innate immunity [28]. Our investigation began with the consistent downregulation of Sema4A observed across multiple atrophy models. Muscle atrophy is driven by an imbalance in protein metabolism, and suppression of Atrogin‐1 and MuRF1 has been shown to protect against muscle loss induced by denervation, glucocorticoids, fasting and tumours [29]. In line with this, we found that AAV‐mediated Sema4A overexpression operates through a synergistic mechanism involving both the suppression of the catabolic atrogene program at baseline and during stress and the active preservation of intrinsic myogenic capacity. Sema4A overexpression robustly activated mTOR phosphorylation, likely shifting the metabolic balance toward synthesis. Interestingly, in psoriatic keratinocytes, decreased Sema4A coincides with elevated mTORC1 signalling [30]. This opposite pattern suggests that Sema4A modulates metabolic homeostasis in a highly context‐dependent manner. Collectively, our results extend the functional landscape of the semaphorin family from the traditional neuroimmune axis to skeletal muscle physiology.

Sema4A exists in both transmembrane and soluble forms and can bind multiple receptors, including Plexin B2, Plexin D1 and Nrp1 [31]. In this study, shRNA screening identified Plexin B2 as the essential mediator for Sema4A in skeletal muscle. The PI3K‐AKT‐mTOR pathway acts as a central regulator determining whether muscle undergoes growth or wasting [6]. Notably, Sema5A has been shown to activate PI3K‐AKT‐mTOR signalling via Plexin receptors, increasing GPX4 synthesis [32], supporting the broader role of semaphorins in metabolic regulation. Consistent with these observations, we position the Sema4A‐Plexin B2 axis upstream of the PI3K‐AKT signalling network. It is well established that in muscle, PI3K‐AKT activation promotes net protein accumulation by suppressing FoxO transcription factors [6, 33]. Our findings indicate that Sema4A engages this pathway to simultaneously inhibit atrogene transcription and sustain mTOR activity, thereby leveraging this classic metabolic mechanism to preserve muscle integrity.

Given that Sema4A functions primarily as an integral transmembrane protein and no soluble form was detected in our system, its spatial engagement with Plexin B2 warrants explanation. As confirmed by several key structural and functional studies [S1–S2], transmembrane semaphorins naturally engage their receptors via cell‐to‐cell contact (*trans*‐interaction) or through lateral associations on the same membrane (*cis*‐interaction). We propose that the lateral *cis‐interaction* between intramuscular Sema4A and Plexin B2 predominantly drives the downstream PI3K‐AKT‐mTOR cascade. This membrane‐restricted modality efficiently regulates muscle metabolism without requiring extracellular release.

Landmark studies have shown that skeletal muscle actively remodels the local immune microenvironment to maintain homeostasis, for example, through secretion of Metrnl to induce M2 macrophage polarization [34]. In parallel, semaphorins and their receptors are known to regulate immunity in clinical settings such as sepsis [28]. Here, we uncover a similar Sema4A‐driven bidirectional crosstalk. To dissect the mechanism, we focused on the metabolic regulation of the secretome. Gdf15, an emerging stress‐responsive myokine secreted directly by muscle fibers [35], has been identified as a core molecule that suppresses proinflammatory signals and coordinates the resolution of inflammation [36]. Given that mTORC1 activates the transcription factor Atf4 [37, 38], which is required for Gdf15 transcription [24], our rescue experiments now indicate that Sema4A‐mediated maintenance of mTOR signalling drives Gdf15 secretion.

Although Gdf15 drives anorexia and catabolism in systemic conditions such as cancer cachexia [26, 39], our findings support its role as a beneficial local stress signal during tissue repair. Consistent with the ability of soluble Sema4A to modulate cytokine production [40], we observed that Sema4A overexpression facilitates a shift of macrophages toward a regenerative M2 phenotype. This macrophage reprogramming relies on intramuscular mTOR activity, as its inhibition blocked M2 polarization and induced a proinflammatory state. Consequently, these alternatively activated macrophages secrete regenerative factors including IL‐4 and IL‐10, thereby supporting regeneration by stimulating proliferation and inhibiting fatty infiltration [12, S3–S5].

## 5 Conclusion and Limitations

In summary, this study identifies a key role for Sema4A in preserving skeletal muscle homeostasis. We show that Sema4A acts through the Plexin B2 receptor to exert dual protective effects by inhibiting FoxO3a‐mediated atrogene transcription while maintaining mTOR signalling. This intracellular regulatory circuit protects against atrophy and fosters a reparative immune microenvironment, potentially involving paracrine factors such as Gdf15. However, limitations should be noted: Our approach relied on AAV‐mediated gene transfer rather than muscle‐specific transgenic models, and we did not functionally block Gdf15 to quantify its specific contribution to the immunomodulatory effects. Despite these constraints, our findings suggest that targeting Sema4A represents a promising therapeutic strategy for treating skeletal muscle atrophy.

## Funding

This work was supported by the Award Funds for Outstanding Young and Middle‐aged Contribution Experts in Health from Fujian Province. The Fujian Provincial Natural Science Foundation (Grant No. 2021J011452 and 2022J011513). The Ningde Municipal Program for the Development of National Clinical Key Specialty Cultivation.

## Ethics Statement

All animals and the experimental protocol conformed to the Animal Welfare Act Guide for Use and Care of Laboratory Animals and were approved by the Institutional Animal Care and Use Committee (IACUC), Fudan University, China.

## Conflicts of Interest

The authors declare no conflicts of interest.

## Supporting information


**Figure S1:** Sema4A is downregulated in muscle atrophy models.
**Figure S2:** Sema4A primes the myogenic program under basal conditions and enhances muscle regeneration following CTX injury.
**Figure S3:** Sema4A preserves myogenic capacity and prevents in vitro myotube atrophy under diverse stress conditions.
**Figure S4:** Sema4A suppresses basal atrogene expression and protects against Dex‐induced skeletal muscle atrophy.
**Figure S5:** Sema4A promotes M2 macrophage polarization and enhances muscle‐immune crosstalk.
**Figure S6:** Sema4A supports a reparative immune microenvironment.

**Table S1:** List of primary antibodies used in this study.
**Table S2:** Primer sequences used for RT‐qPCR.
**Table S3:** siRNA sequences used in this study.

## Data Availability

All data in this study are provided in the paper and Supporting Information and Tables.
