# Supplementary material for: Sema4A Protects Against Muscle Atrophy and Promotes Repair by Regulating Intracellular Metabolic Signalling
Source: J Cachexia Sarcopenia Muscle. 2026 May 29;17(3):e70315. doi: 10.1002/jcsm.70315 (PMC13240067; doi:10.1002/jcsm.70315)
Supplement: Supplementary file 1 — Figure S1: Sema4A is downregulated in muscle atrophy models. Figure S2: Sema4A primes the myogenic program under basal conditions and enhances muscle regeneration following CTX injury. Figure S3: Sema4A preserves myogenic capacity and prevents in vitro myotube atrophy under diverse stress conditions. Figure S4: Sema4A suppresses basal atrogene expression and protects against Dex‐induced skeletal muscle atrophy. Figure S5: Sema4A promotes M2 macrophage polarization and enhances muscle‐immune crosstalk. Figure S6: Sema4A supports a reparative immune microenvironment. [file JCSM-17-e70315-s002.docx]

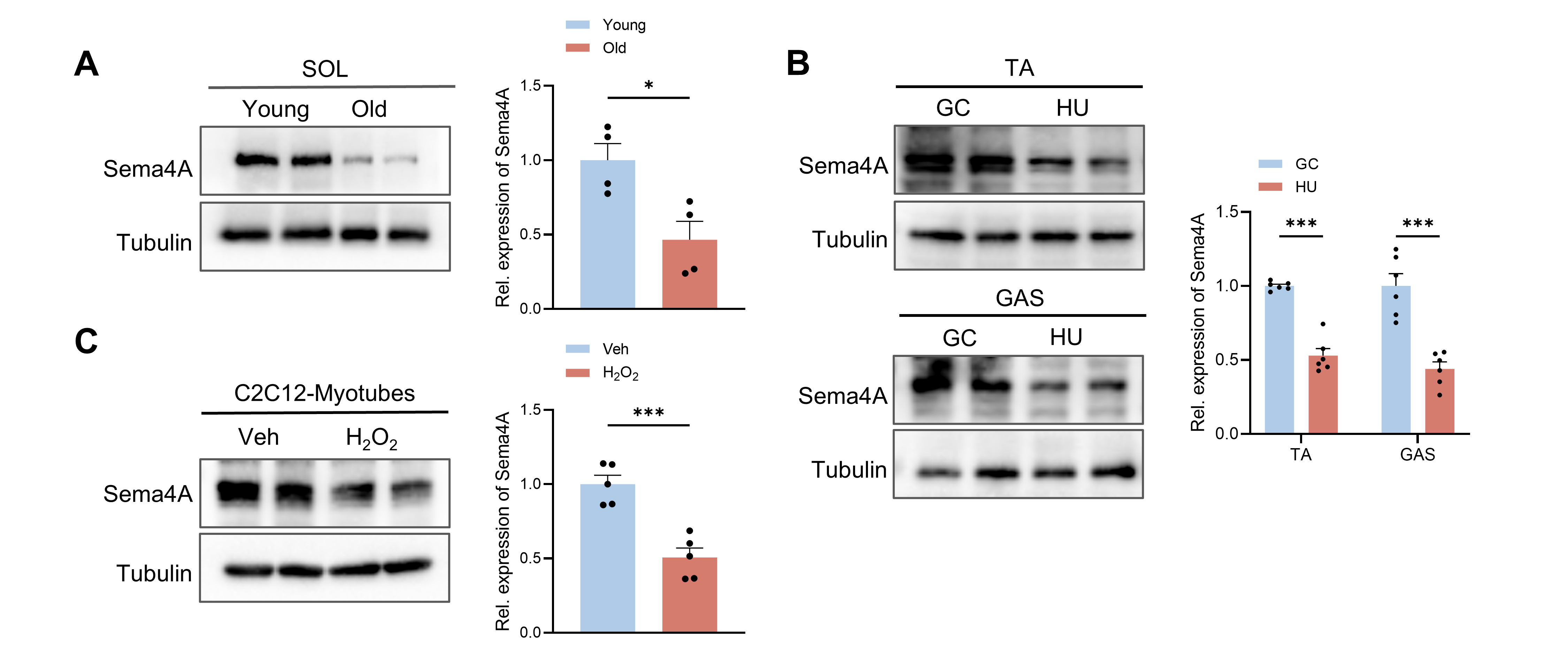


**Figure S1. Sema4A is downregulated in muscle atrophy models.** (**A**) Validation of Sema4A protein expression in the soleus (SOL) muscle of aged mice (18 months) (*n* = 4). (**B**) Validation of Sema4A protein expression in TA and gastrocnemius (GAS) muscles following hindlimb immobilization (*n* = 6). (**C**) Validation of Sema4A protein expression in an H_2_O_2_-induced C2C12 myotube atrophy model (*n* = 5). GC, ground control, HU, hindlimb unloading. ^*^*p <* 0.05; ^***^*p <* 0.001. Data are presented as Mean ± SEM. Statistical significance for two-group comparisons was determined using an unpaired two-tailed Student's *t*-test, while multiple parallel indicators were evaluated using multiple *t*-tests.


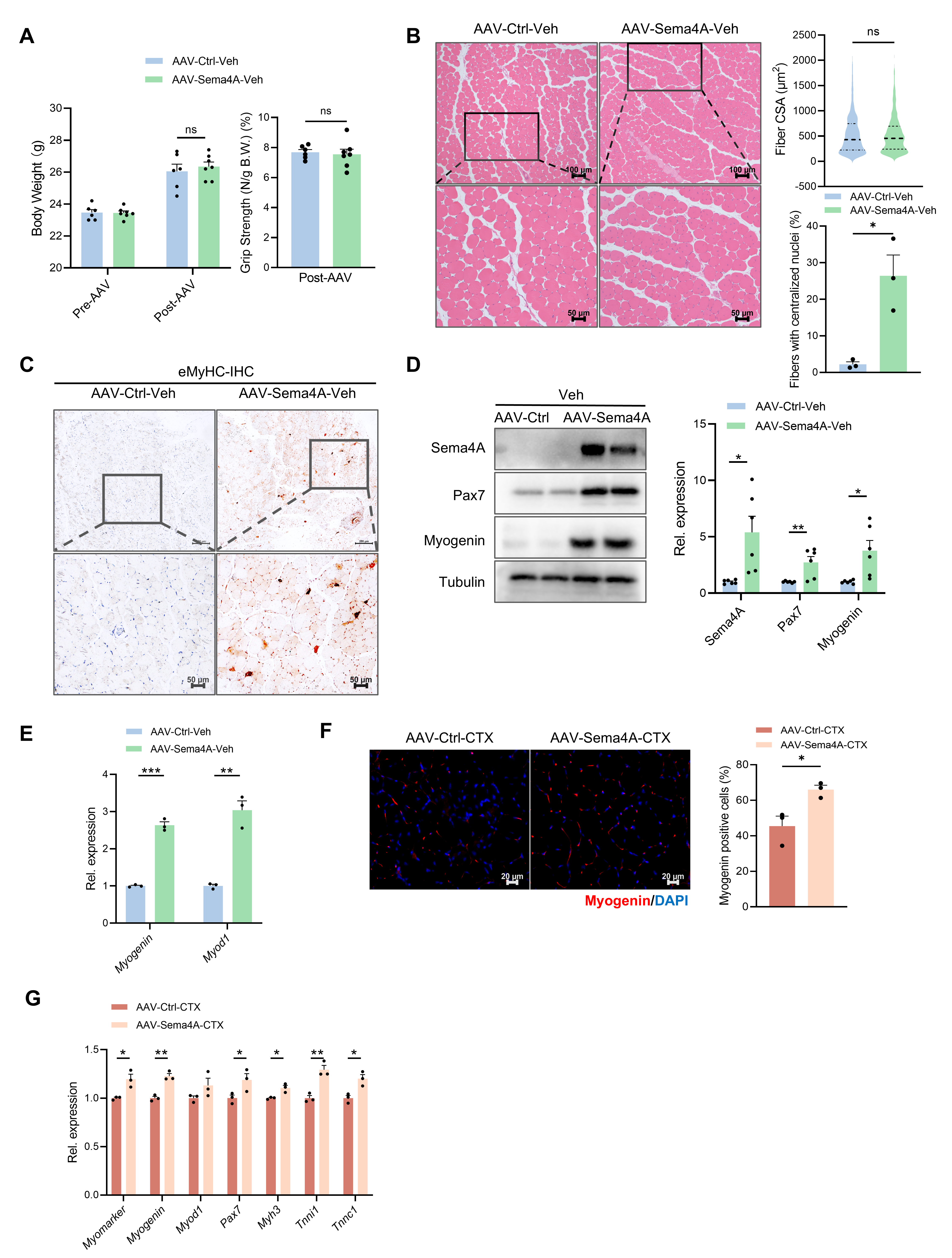


**Figure S2. Sema4A primes the myogenic program under basal conditions and enhances muscle regeneration following CTX injury.** (**A**) Body weight changes before and 21 days after AAV injection (left), and forelimb grip strength evaluation (right) under basal conditions (*n* = 6-7). (**B**) Representative H&E staining of uninjured TA muscles. Quantitative analyses of myofiber CSA (300-400 myofibers were analyzed per image) and the percentage of centrally nucleated fibers are shown (*n* = 3). Scale bars, 100 μm /50 μm. (**C**) Representative immunofluorescence staining for eMyHC in uninjured TA muscles. Scale bars, 200 μm and 50 μm. (**D**) Representative Western blot images and quantification of myogenic markers (Pax7 and Myogenin) in AAV-Ctrl and AAV-Sema4A muscles under basal conditions (*n* = 6). (**E**) Relative mRNA expression of *Myogenin* and *Myod1* under basal conditions, determined by RT-qPCR (*n* = 3). (**F**) Representative immunofluorescence staining for Myogenin (red) and DAPI (blue) in TA muscles following CTX injury. Quantification of the percentage of Myogenin-positive cells (*n* = 3). Scale bars: 20 μm. (**G**) Relative mRNA expression of indicated muscle regeneration genes in TA muscles following CTX injury, determined by RT-qPCR (*n* = 3). ^*^*p <* 0.05; ^*^*^*^p <* 0.01; ^***^*p <* 0.001. Data are presented as Mean ± SEM. Statistical significance for two-group comparisons was determined using an unpaired two-tailed Student's *t*-test, while multiple parallel indicators were evaluated using multiple *t*-tests.


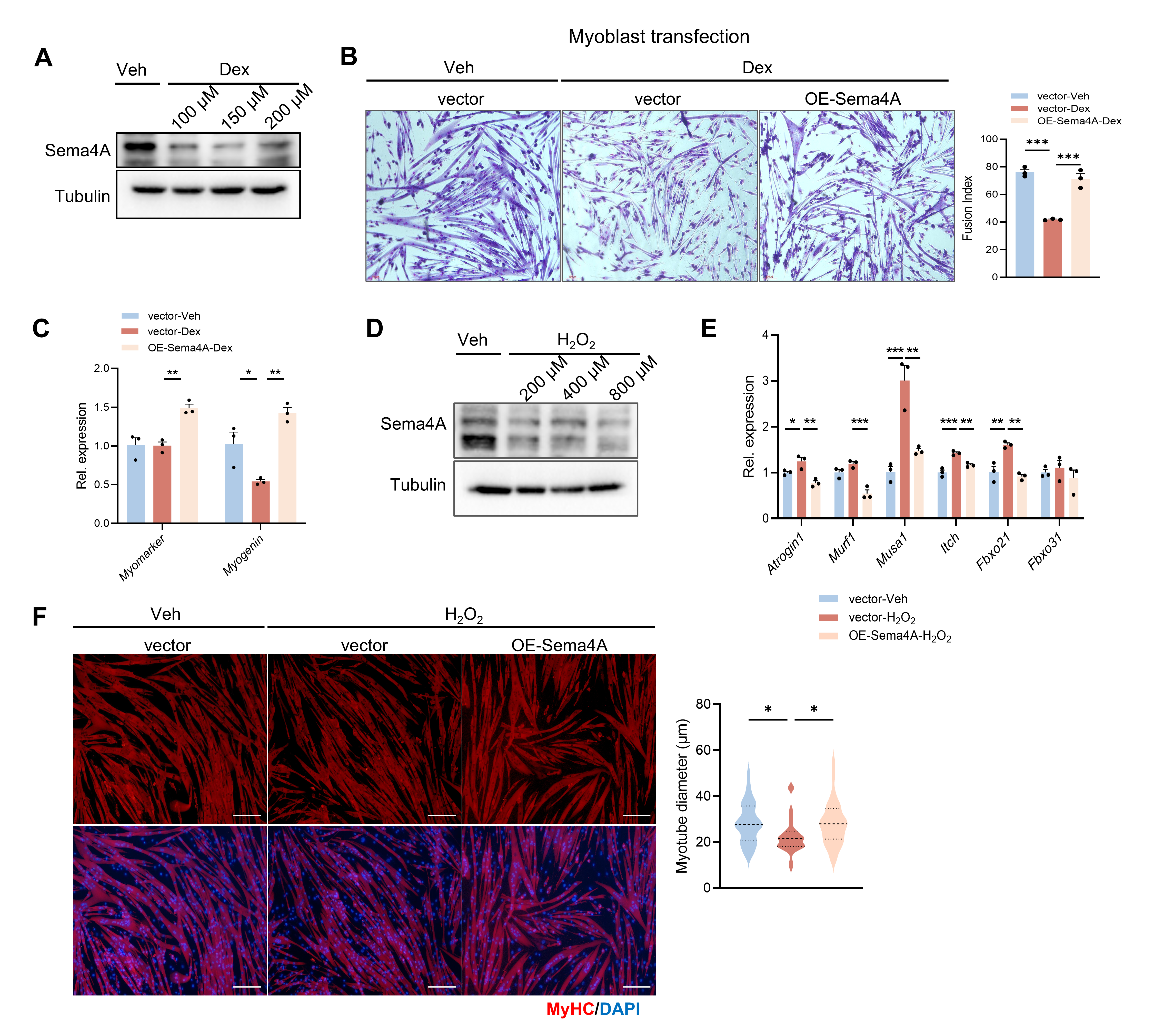


**Figure S3.** **Sema4A preserves myogenic capacity and prevents *in vitro* myotube atrophy under diverse stress conditions.** (**A**) Western blot analysis of Sema4A expression in C2C12 myotubes treated with increasing concentrations of Dex. (**B**) Representative Giemsa staining images of C2C12 myoblasts under the indicated conditions. The fusion index was quantified (*n* = 3). Scale bar: 100 μm. (**C**) Relative mRNA expression of *Myomarker* and *Myogenin* in C2C12 myotubes under the indicated conditions. (*n* = 3). (**D**) Western blot analysis of Sema4A expression in C2C12 myotubes treated with increasing concentrations of H_2_O_2_. (**E**) Relative mRNA expression of indicated atrophy-related genes in C2C12 myotubes treated with H_2_O_2_ under the indicated conditions. (*n* = 3). (**F**) Representative MyHC immunofluorescence images of C2C12 myotubes under the indicated conditions. Quantification of myotube diameter is shown. Scale bar: 100 μm. *n* = 30 myotubes per condition. ^*^*p <* 0.05; ^*^*^*^p <* 0.01; ^***^*p <* 0.001. Data are presented as Mean ± SEM. Multiple groups were evaluated via one-way ANOVA.


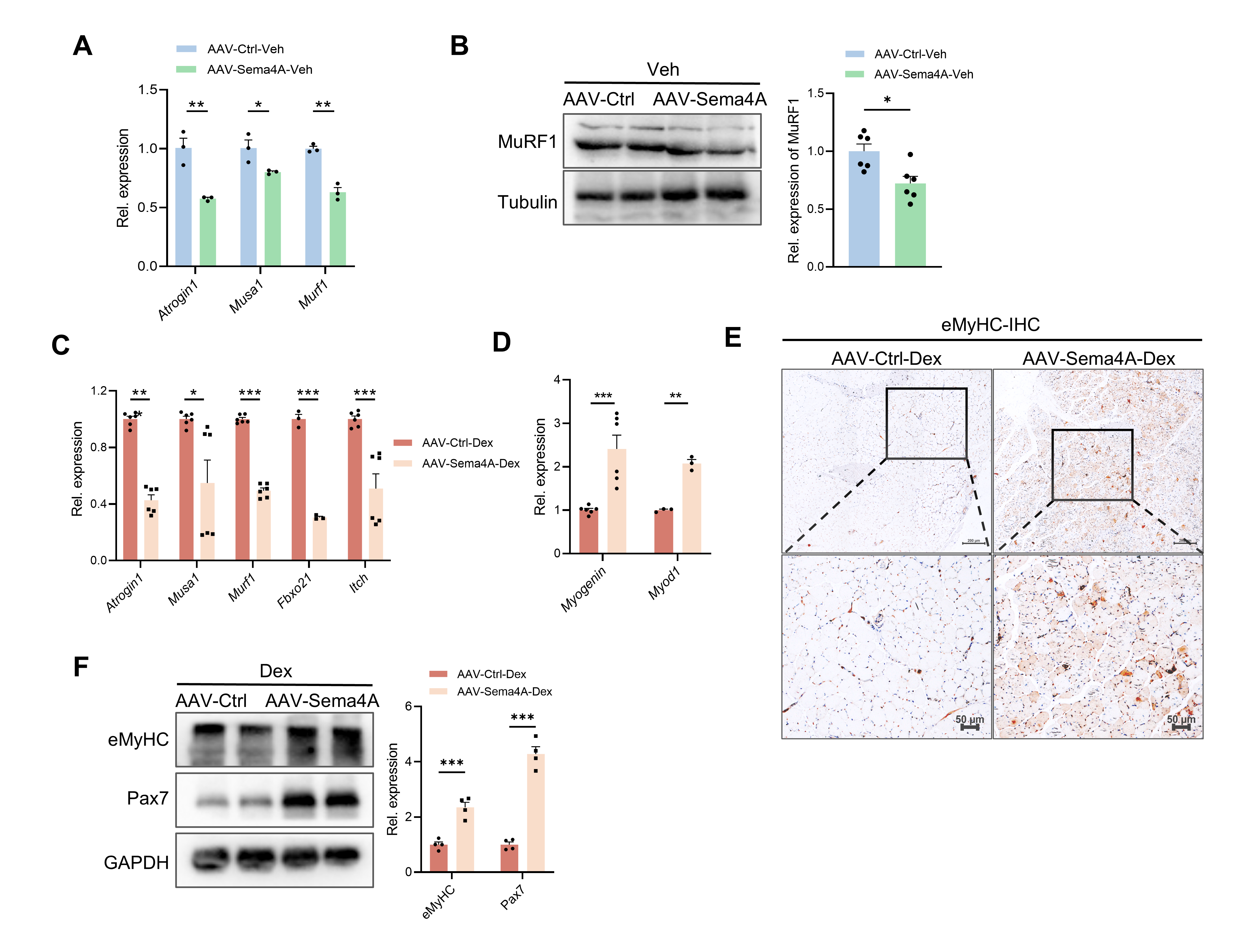


**Figure S4.** **Sema4A suppresses basal atrogene expression and protects against Dex-induced skeletal muscle atrophy. (A)** Relative mRNA expression of indicated atrophy-related genes in TA muscles following AAV injection under basal conditions (in the absence of Dex treatment), determined by RT-qPCR (*n* = 3). **(B)** Western blot analysis detailing the basal protein expression of MuRF1 in TA muscles without Dex treatment (*n* = 6). **(C)** Relative mRNA expression of indicated atrophy-related genes in TA muscles following Dex-induced atrophy, determined by RT-qPCR (*n* = 3-6). **(D)** Relative mRNA expression of the myogenic genes *Myogenin* and *Myod1* in TA muscles subjected to Dex treatment, determined by RT-qPCR (*n* = 3-6). **(E)** Representative immunohistochemistry images for eMyHC in muscle cross-sections from Dex-treated mice. Scale bars, 200 μm and 50 μm. **(F)** Western blot analysis and quantification of eMyHC and Pax7 expression in TA muscles from AAV-Ctrl-Dex and AAV-4A-Dex mice (*n* = 4). ^*^*p <* 0.05; ^*^*^*^p <* 0.01; ^***^*p <* 0.001. Data are presented as Mean ± SEM. Statistical significance for two-group comparisons was determined using an unpaired two-tailed Student's *t*-test, while multiple parallel indicators were evaluated using multiple *t*-tests.


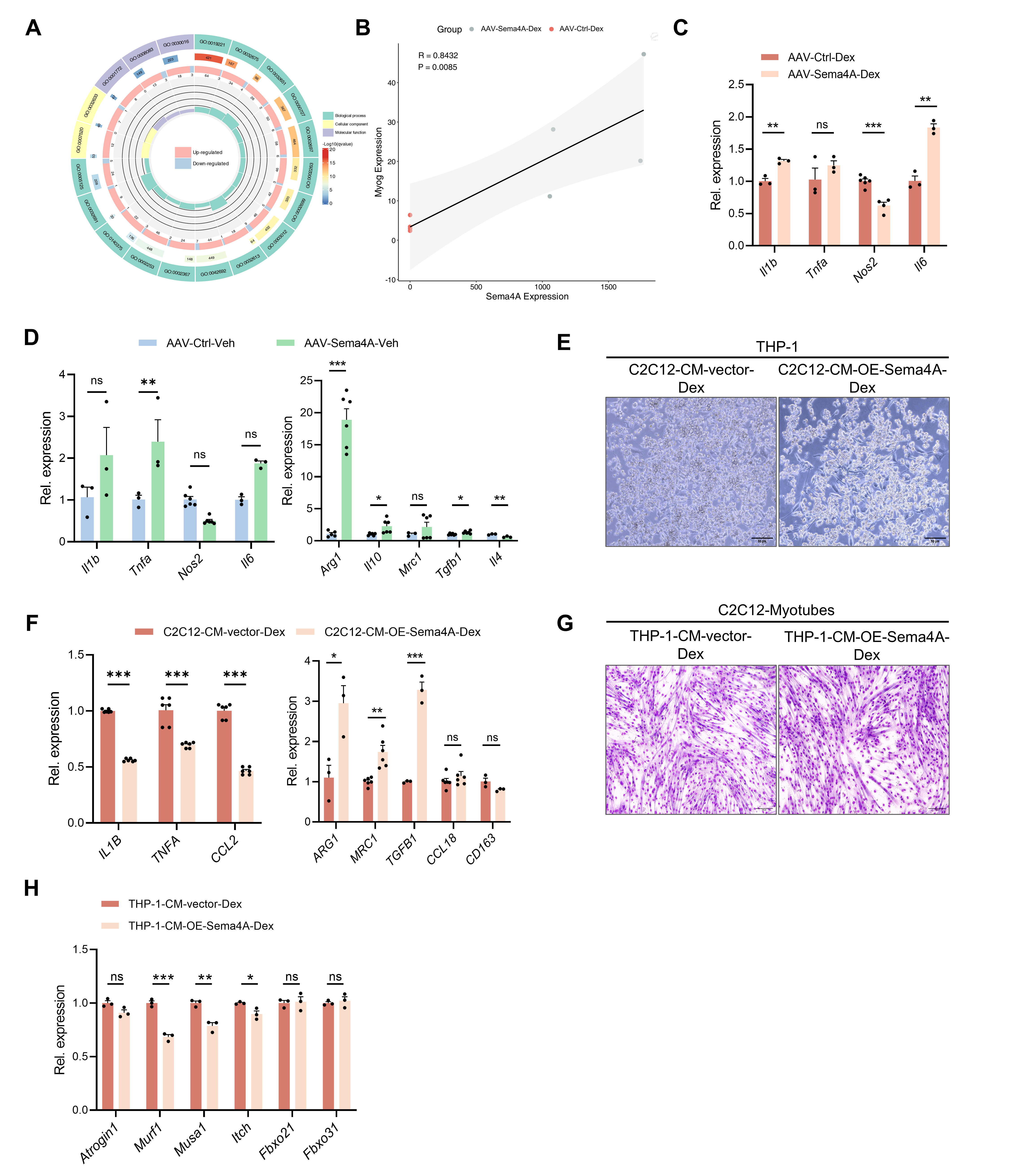


**Figure S5.** **Sema4A promotes M2 macrophage polarization and enhances muscle-immune crosstalk.** (**A**) Circle plot illustrating GO enrichment analysis. (**B**) Correlation analysis between *Sema4A* and *Myog (Myogenin)* in the RNA-seq dataset. **(C)** Relative mRNA expression of indicated M1 macrophage markers in TA muscles from AAV-Ctrl-Dex and AAV-Sema4A-Dex mice determined by RT-qPCR (*n* = 3). **(D)** Relative mRNA expression of indicated M1/M2 macrophage markers in TA muscles following AAV injection in the absence of Dex treatment (*n* = 3-6). **(E)** Representative images showing the morphology of THP-1 cells treated with CM derived from C2C12-OE-Sema4A-Dex myotubes. **(F)** Relative mRNA expression of indicated M1/M2 markers in THP-1 cells following 48 h of incubation with the indicated C2C12-derived CM (diluted 1:1 with fresh medium) (*n* = 3-6). **(G)** Representative images showing the morphology of C2C12 myotubes treated with CM from the pre-treated THP-1 cells. **(H)** Relative mRNA expression of atrophy-related genes in C2C12 myotubes treated with CM from the pre-treated THP-1 cells (*n* = 3). ^*^*p <* 0.05; ^*^*^*^p <* 0.01; ^***^*p <* 0.001. Data are presented as Mean ± SEM. Significance between two groups was determined by an unpaired two-tailed Student's *t*-test, or multiple *t*-tests for parallel indicators.


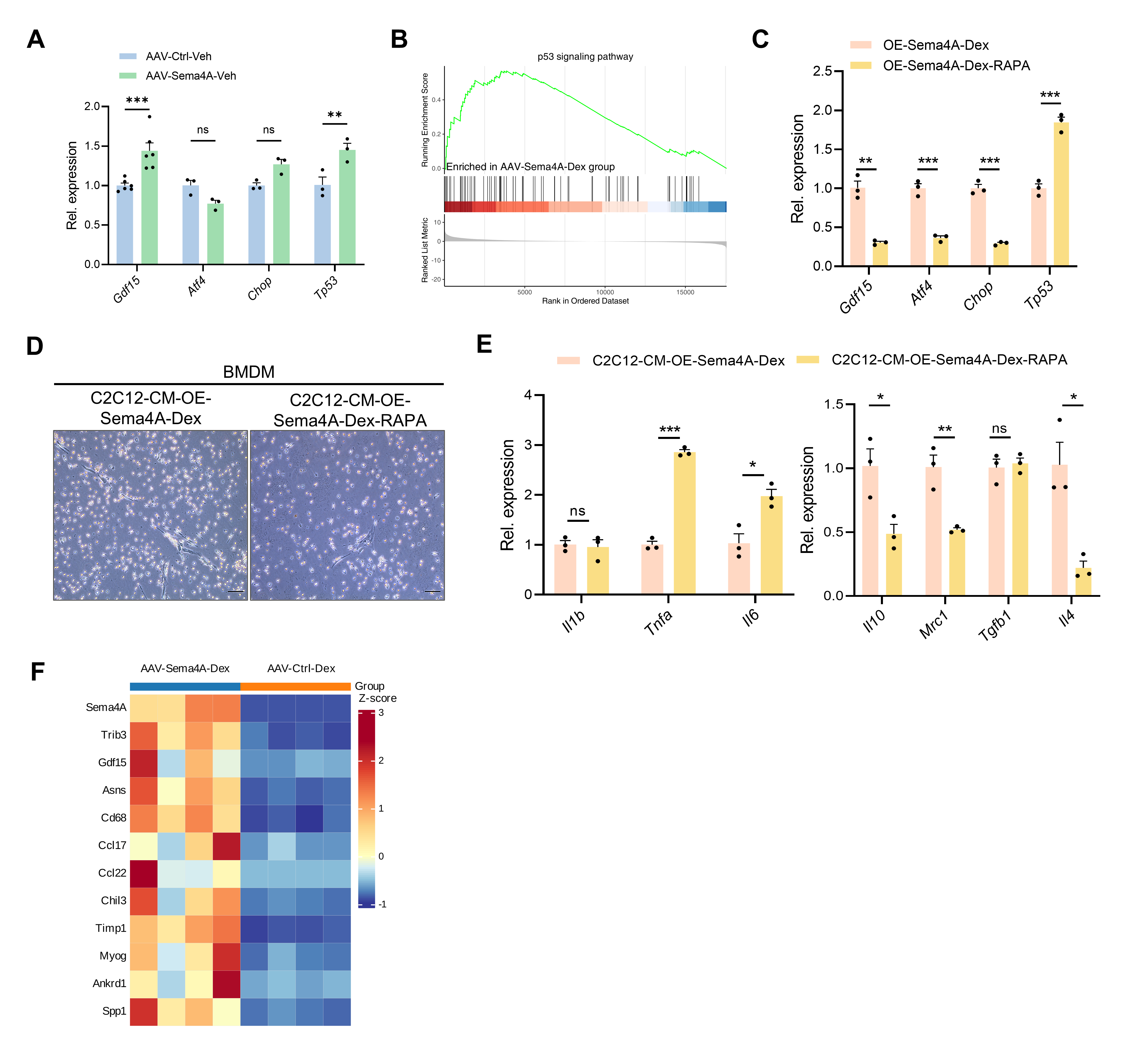


**Figure S6. Sema4A supports a reparative immune microenvironment.** **(A)** Relative mRNA expression of *Gdf15*, *Atf4*, *Chop*, and *Tp53* in TA muscles following AAV injection under basal conditions (in the absence of Dex treatment), determined by RT-qPCR (*n* = 3-6). **(B)** Gene Set Enrichment Analysis (GSEA) of the p53 signaling pathway. **(C)** Relative mRNA expression of *Gdf15*, *Atf4*, *Chop*, and *Tp53* in Sema4A-overexpressing myotubes treated with or without the mTOR inhibitor Rapamycin (250 nM, 24 h) (*n* = 3). **(D)** Representative bright-field images displaying the morphology of BMDMs cultured with the indicated CM. Note the reduction of elongated M2-like cells upon mTOR inhibition. Scale bars, 50 μm. **(E)** Relative mRNA expression of M1/M2 markers in BMDMs cultured with the indicated CM, determined by RT-qPCR (*n* = 3). **(F)** Heatmap displaying genes associated with Sema4A-driven Gdf15 secretion, facilitating macrophage recruitment and M2 polarization to accelerate muscle repair. ^*^*p <* 0.05; ^*^*^*^p <* 0.01; ^***^*p <* 0.001. Data are presented as Mean ± SEM. Comparisons between two groups across multiple parallel indicators were evaluated using multiple *t*-tests.
