# Supplementary material for: Sema4A Protects Against Muscle Atrophy and Promotes Repair by Regulating Intracellular Metabolic Signalling
Source: J Cachexia Sarcopenia Muscle. 2026 May 29;17(3):e70315. doi: 10.1002/jcsm.70315 (PMC13240067; doi:10.1002/jcsm.70315)
Supplement: Supplementary file 2 — Table S1: List of primary antibodies used in this study. Table S2: Primer sequences used for RT‐qPCR. Table S3: siRNA sequences used in this study. [file JCSM-17-e70315-s001.docx]

**Supplementary materials and methods**

**Antibodies**

All antibodies are listed in **Table S1.**

**Western blot analysis**

Samples were lysed in RIPA buffer (Beyotime) supplemented with 1% protease inhibitor, 1% PMSF, and 0.5% phosphatase inhibitor (APExBIO, Houston, TX, USA). Lysates were clarified by centrifugation at 12,000 × *g* for 15 minutes at 4°C, and protein concentrations were determined using the BCA assay (23227, Thermo Fisher). Equal amounts of protein (20-30 μg) were denatured in LDS sample buffer at 95°C for 10 minutes, separated by SDS-PAGE, and transferred onto nitrocellulose membranes. Membranes were blocked with 5% skim milk in TBST and incubated with primary antibodies overnight at 4°C. Following TBST washes, HRP-conjugated secondary antibodies were applied for 2 hours at room temperature. Protein bands were visualized using a chemiluminescence system (Bio-Rad) and quantified with Image Lab software.

**Immunoprecipitation (IP)**

Whole cell lysates were harvested from C2C12 myotubes using lysis buffer. For immunoprecipitation, 1.0-3.0 mg of total protein lysate was incubated with 0.5-4.0 µg of anti- Plexin B2 antibody (10602-1-AP, Proteintech) and Protein A/G-PLUS Agarose beads (sc-2003, Santa Cruz). The immune complexes were washed and subsequently analyzed by immunoblotting.

**Gene expression analysis**

Total RNA was extracted using TRIzol reagent (9109, Takara). cDNA synthesis was performed using the Hifair AdvanceFast cDNA Synthesis kit (11149ES60, Yeasen). Quantitative Real-time PCR (qPCR) was conducted using SYBR Green Master Mix (10102ES08, Yeasen). Primer sequences are listed in **Table S2**.

**Supplementary references**

S1. Haklai-Topper, L., et al., Cis interaction between Semaphorin6A and Plexin-A4 modulates the repulsive response to Sema6A. *The EMBO Journal*, 2010. 29(15): p. 2635-45.

S2. Rozbesky, D., et al., Structural basis of semaphorin‐plexin cis interaction. *The EMBO Journal*, 2020. 39(13): p. EMBJ2019102926.

S3. Kistner, T.M., B.K. Pedersen, and D.E. Lieberman, Interleukin 6 as an energy allocator in muscle tissue. *Nature Metabolism*, 2022. 4(2): p. 170-179.

S4. Chen, W., et al., Bidirectional roles of skeletal muscle fibro-adipogenic progenitors in homeostasis and disease. *Ageing Research Reviews*, 2022. 80: p. 101682.

S5. Severinsen, M.C.K. and B.K. Pedersen, Muscle-Organ Crosstalk: The Emerging Roles of Myokines. *Endocr Rev*, 2020. 41(4): p. 594-609.

**Table S1. List of primary antibodies used in this study.**

| Antibodies | Source | Identifier |
| --- | --- | --- |
| Myogenin | Santa Cruz | sc-52903 |
| Myostatin | Proteintech | 19142-1-AP |
| MuRF1(Trim63) | Proteintech | 55456-1-AP |
| Atrogin1(Mafbx) | Abclonal | A6825 |
| FoxO3a | Proteintech | 66428-1-Ig |
| Plexin B2 | Proteintech | 10602-1-AP |
| Sema4A | Origene | TA381347 |
| Laminin | Abcam | ab11575 |
| GDF15 | Abclonal | A0185 |
| Pax7 | Santa Cruz | sc-81648 |
| MyHC | Santa Cruz | sc-376157 |
| eMyHC | Developmental Studies HybridomaBank | F1.652 |
| normal Rabbit IgG | Cell Signaling Technology | 2729 |
| CD68 | Invitrogen | 14-0688-82 |
| CD206 | Cell Signaling Technology | 24595 |
| Phospho-PI3-kinase p85- alpha/ gamma (Tyr467/199) pAb | Abmart | T40116 |
| [PI3-kinase p85-alpha/gamma pAb](https://www.ab-mart.com.cn/page.aspx?node=%2077%20&id=%201221) | Abmart | [T40064](https://www.ab-mart.com.cn/page.aspx?node=%2077%20&id=%201221) |
| Phospho-Akt (Thr450) Antibody | Selleck | J1B12 |
| AKT | Selleck | E16N17 |
| mTOR | Selleck | F0169 |
| Phospho-mTOR (Ser2448) | Cell Signaling Technology | 5536 |
| Tubulin | Proteintech | 66240-1-lg |

**Table S2. Primer sequences used for RT-qPCR.**

| Primer name | Primer sequence (5´ to 3´) |
| --- | --- |
| Mouse_*Mafbx*_F | AGTGAGGACCGGCTACTGTG |
| Mouse_*Mafbx*_R | GATCAAACGCTTGCGAATCT |
| Mouse_*Murf1*_F | TGACATCTACAAGCAGGAGTGC |
| Mouse_*Murf2*_R | TCGTCTTCGTGTTCCTTGC |
| Mouse_*Fbxo31*_F | AGACATCTTCCACGAGCACA |
| Mouse_*Fbxo31*_R | GGTAGGTCAGGCAGTTGTCG |
| Mouse_*Itch*_F | AACAACGCCTTAACCCTAAGAA |
| Mouse_*Itch*_R | CATGCCCAGCTTGTACTGTTAC |
| Mouse_*Musa1*_F | TTTCAACTGTGAGACTGAATTGC |
| Mouse_*Musa1*_R | TTTCTACCTTTTGGCAGAAATAAAG |
| Mouse_*Fbxo21*_F | CTTGAACCTCTACATGCACCAG |
| Mouse_*Fbxo21*_R | AAGTGGCTTGGGAAGTTGAC |
| Mouse_*Actin*_F | GGCTGTATTCCCCTCCATCG |
| Mouse_*Actin*_R | CCAGTTGGTAACAATGCCATGT |
| Mouse_*Myogenin*_F | CCCACAATCTGCACTCCCTT |
| Mouse_*Myogenin*_R | CACCCAGCCTGACAGACAAT |
| Mouse_*Myod1*_F | GCTACGACACCGCCTACTAC |
| Mouse_*Myod1*_R | GAGATGCGCTCCACTATGCT |
| Mouse_*Pax7*_F | TCTCCAAGATTCTGTGCCGAT |
| Mouse_*Pax7*_R | CGGGGTTCTCTCTCTTATACTCC |
| Mouse_*Myh3*_F | GGCCAAACTGATCACTCGGA |
| Mouse_*Myh3*_R | ATGGACTCCCTCCTCTGCAT |
| Mouse_*Myomaker*_F | CCTGCTGTCTCTCCCAAG |
| Mouse_*Myomaker*_R | AGAACCAGTGGGTCCCTAA |
| Mouse_*Tnnc1*_F | GCAGGAGATGATTGACGAAGTAGA |
| Mouse_*Tnnc1*_R | CGAACCATCATGACAAGAAACTCA |
| Mouse_*Tnni1*_F | CGAACCATCATGACAAGAAACTCA |
| Mouse_*Tnni1*_R | TTACCTCTCGGAGCGCATTC |
| Mouse_*Sema4A*_F | AAGCCCCTTTGACCCTGTTC |
| Mouse_*Sema4A*_R | AGACTCGCTCAATGTCCGTG |
| Mouse_*Gdf15*_F | ACTCAGGACACAAGCGACATGG |
| Mouse_*Gdf15*_R | AGCAGCAGGAACAGCAGGAAC |
| Mouse_*Atf4*_F | AAGGAGGATGCCTTTTCCGG |
| Mouse_*Atf4*_R | TCCAACGTGGTCAAGAGCTC |
| Mouse_*Chop*_F | AGTGGCACAGCTAGCTGAAG |
| Mouse_*Chop*_R | TCCGGAGAGACAGACAGGAG |
| Mouse_*Tp53*_F | TTCATTGGGACCATCCTGGC |
| Mouse_*Tp53*_R | TGGGAAGGAGGAGGATGAGG |
| Mouse_*Plxnb2*_F | TCAATGGCACTCACCTGGAC |
| Mouse_*Plxnb2*_R | ACACACTGTAGCTGTGCTCC |
| Mouse_*Il6*_F | GGAGCCCACCAAGAACGATAGTC |
| Mouse_ *Il6*_R | TCACCAGCATCAGTCCCAAGAAG |
| Mouse_*Arg1*_F | CTCCAAGCCAAAGTCCTTAGAG |
| Mouse_*Arg1*_R | AGGAGCTGTCATTAGGGACATC |
| Mouse_*Il10*_F | GCTCTTACTGACTGGCATGAG |
| Mouse_*Il10*_R | CGCAGCTCTAGGAGCATGTG |
| Mouse_*Mrc1*_F | CTCTGTTCAGCTATTGGACGC |
| Mouse_*Mrc1*_R | CGGAATTTCTGGGATTCAGCTTC |
| Mouse_*Il4*_F | GGTCTCAACCCCCAGCTAGT |
| Mouse_*Il4*_R | GCCGATGATCTCTCTCAAGTGAT |
| Mouse_*Tgfb1*_F | CTCCCGTGGCTTCTAGTGC |
| Mouse_*Tgfb1*_R | GCCTTAGTTTGGACAGGATCTG |
| Mouse_*Il1b*_F | GCAACTGTTCCTGAACTCAACT |
| Mouse_*Il1b*_R | ATCTTTTGGGGTCCGTCAACT |
| Mouse_*Tnfa*_F | CTGAACTTCGGGGTGATCGG |
| Mouse_*Tnfa*_R | GGCTTGTCACTCGAATTTTGAGA |
| Mouse_*Nos2*_F | GTTCTCAGCCCAACAATACAAGA |
| Mouse_*Nos2*_R | GTGGACGGGTCGATGTCAC |
| Human_*IL1B*_F | CCACCTCCAGGGACAGGATA |
| Human_*IL1B*_R | TCAACACGCAGGACAGGTAC |
| Human_*TNFA*_F | CTTCCAGCTGGAGAAGGGTG |
| Human_*TNFA*_R | CCCAAAGTAGACCTGCCCAG |
| Human_*CCL2*_F | GACCATTGTGGCCAAGGAGA |
| Human_*CCL2*_R | TTGGGTTTGCTTGTCCAGGT |
| Human _*ARG1*_F | AAGATTCCCGATGTGCCAGG |
| Human _ARG1_R | GTCCACGTCTCTCAAGCCAA |
| Human_*TGF-β1*_F | GACTTTTCCCCAGACCTCGG |
| Human_*TGF-β1*_R | ATAGGGGATCTGTGGCAGGT |
| Human_*MRC1*_F | GCCTCGTTGTTTTGCGTCTT |
| Human_*MRC1*_R | GAGAACAGCACCCGGAATGA |
| Human_*CD163*_F | GCGGGAGAGTGGAAGTGAAA |
| Human_*CD163*_R | ACCTGCACTGGAATTAGCCC |
| Human_*CCL18*_F | TGAAGCTGAATGCCTGAGGG |
| Human_*CCL18*_R | GGGCATAGCAGATGGGACTC |

**Table S3. siRNA sequences used in this study.**

| Gene | siRNA sequence (5´ to 3´) |
| --- | --- |
| Mouse-*Sema4A*-sense | CGCAUCUACGCAGUCUUUTT |
| Mouse-*Sema4A*-antisense | AAAGACUGCGUAGAUGCGGTT |
| *Negative*-sense | UUCUCCGAACGUGUCACGUTT |
| *Negative*-antisense | ACGUGACACGUUCGGAGAATT |
